# Important Aspects of the Design of Experiments and Data Treatment in the Analytical Quality by Design Framework for Chromatographic Method Development

**DOI:** 10.3390/molecules29246057

**Published:** 2024-12-23

**Authors:** Bianca F. G. Passerine, Márcia C. Breitkreitz

**Affiliations:** Institute of Chemistry, University of Campinas (UNICAMP), Campinas 13083-970, SP, Brazil; b265998@dac.unicamp.br

**Keywords:** liquid chromatography, analytical quality by design, experimental design, MODR

## Abstract

In the analytical quality by design (AQbD) framework, the design of experiments (DOE) plays a very important role, as it provides information about how experimental input variables influence critical method attributes. Based on the information obtained from the DOE, mathematical models are generated and used to build the method operable design region (MODR), which is a robust region of operability. Data treatment steps are usually carried out in software such as Fusion QbD, Minitab, or StaEase 360, among others. Although there are many studies in the literature that use the DOE, none of them address important aspects of data treatment for optimization and MODR generation and compare different software calculations. The purpose of this study is to contribute to a better understanding of data treatment aspects that are frequently misread or not fully understood, such as model selection, ANOVA results, and residual analysis. The discussion will be guided by the separation of curcuminoids, using ultra-high performance liquid chromatography and eight quality attributes as responses. This study highlights the importance of carefully selecting and evaluating models, as they significantly influence the generation of the MODR. Moreover, the findings emphasize that it is essential to incorporate uncertainties into the contour plots to accurately determine the MODR in compliance with the ICH Q14 guidelines and USP General Chapter <1220>.

## 1. Introduction

Liquid chromatography (LC) is the most widely used separation technique supporting drug development activities and product quality control. The separation of analytes through LC predominantly depends on intrinsic mobile phase parameters, such as polarity, pH, flow rate, and composition, as well as the inherent properties of the sample matrix, type of stationary phase, type of detector, and environmental factors, such as temperature. For many years, the development of LC methods has been carried out using the so-called “one factor at a time” (OFAT) approach, which consists of testing different operating conditions by varying one factor at a time while keeping the other variables constant and requires a large number of experiments. This strategy can be misleading because potential interactions between variables are not investigated. Also, the OFAT approach does not facilitate the execution of possible modifications that may be needed in the future since it does not provide an overview of the response’s behavior within the experimental domain [1]. An enhanced strategy for developing LC methods known as “Analytical Quality by Design (AQbD)” has recently gained attention, especially in pharmaceutical industrial environments. Even though the AQbD strategy is not limited to LC method development, the concept was developed based on this technique due to its wide use in the pharmaceutical industry [2].

AQbD is a systematic approach to analytical method development that emphasizes method understanding and control based on sound science and quality risk management. The AQbD concept has become a trending topic over the past decade, especially after the release of the US Pharmacopeia General Chapter (1220) [3] and the International Council of Harmonization ICH Q14 guideline [4]. These documents highlighted a science and risk-based approach to developing and maintaining analytical procedures suitable for the quality assessment of drug substances and drug products. The AQbD framework consists of four main steps, starting with the definition of the analytical target profile (ATP), which describes the purpose of the method and specifies the quality requirements the method must fulfill. Attributes that are critical for appropriate chromatographic method performance, such as retention factor, resolution, and tailing factor, are also identified in this step and referred to as critical method attributes (CMA) [2]. The second stage involves a risk assessment to attribute a risk (low, medium, high) to each experimental variable (such as temperature, pH, flow rate, etc.) on the CMAs. The high-risk variables are called critical method parameters (CMPs) and should be studied as a function of CMAs using multivariate methods—this is where the design of experiments (DOE) plays a central role within the AQbD approach [5].

The DOE provides high-quality information about the effects of the CMPs on the attributes of the method performance and, therefore, can be applied to the development of analytical methods [6]. This is usually carried out in two steps: screening and optimization. The screening step is required when no or very little information about the method is available, and it involves the study of primary chromatographic variables that are likely to present a high influence on the CMAs and provide a direction for their levels. In this step, it is common to study the stationary phase chemistry (“column type”), the type of organic modifier (protic, aprotic, basic), the range of pH (acidic; basic, neutral), the presence of ionic pairing, etc. This provides the basic information for the method development and can be accomplished by full factorial designs, fractional factorial designs, and optimal designs [7]. It should be noted that the design must be chosen to allow the calculation of (at least) linear first-order effects without bias to provide accurate guidance in this step; therefore, care should be taken with low-resolution (i.e., Res III) and saturated designs.

In the optimization step, some of the primary factors are examined in more detail, and secondary variables are included, as well, to determine the best overall analytical conditions [1]. The responses acquired from the experiments are modeled as a function of CMPs using statistical approaches to develop prediction models for the CMAs. Therefore, the DOE methods include those that are capable of estimating linear interactions and higher-order terms, if necessary. Examples include central composite design (CCD), Box Behnken (BB), Doehlert, and optimal designs. In this stage, the goodness of fit and prediction capacity are fundamental aspects of the mathematical models; therefore, a rigorous statistical examination of the models derived from the DOE using tools such as analysis of variance (ANOVA), the evaluation of important coefficients to avoid overfitting, and, as a consequence, misleading models, and analysis of residuals must be performed for the further generation of a reliable design space [8] or method operable design region (MODR) [9].

Since the MODR is built considering all CMAs simultaneously, it can be established using multi-response optimization tools, such as graphical optimization and desirability functions. In the former, the contour plot of all responses is overlaid to estimate the intersection region, whereas, in the latter, multiple responses are transformed into a single global response obtained using the geometric mean [10]. An important aspect is that the MODR should be computed with uncertainty boundaries, which can be achieved using confidence, prediction, or tolerance intervals (the latter two are preferred) with Monte Carlo simulations associated with capability indexes such as *C_pk_* [8] or propagation of error (POE) [11]. The MODR is considered a robust working region since experimental variations within this region do not cause the failure of any CQA. Thus, robustness is incorporated during development rather than being evaluated at the validation step [12].

Usually, all stages from DOE planning to the construction of the MODR are performed using software such as Fusion QbD (S-Matrix, Eureka, CA, USA), Design Expert or Statease 360 (Stat-Ease Inc., Minneapolis, MN, USA), Minitab (Minitab, State College, PA, USA), or Statistica (StatSoft, Hamburg, Germany), among others. Among these software programs, Fusion QbD stands out for being an automated experimentation platform that is integrated with multiple chromatographic data software (CDS), such as Empower, which allows for automated bi-directional data exchange, thereby eliminating errors and reducing the user’s manual transcription work associated with manual data transfer. This was a milestone for the use of the design of experiments (DOE) in analytical development [13]. Nevertheless, several other software can be used, such as those mentioned above, which perform data treatment in an offline mode. Calculations and available tools may differ from one software to the other; therefore, one of the goals of this paper is to compare the outcomes of two commonly used software: Design Expert and Fusion QbD.

Even though many papers report the use of the DOE in separation techniques [6,14,15,16,17,18,19,20], a practical approach for data interpretation and the discussion of important topics found in software outcomes are not found in the literature. This paper aims to contribute to understanding some important features of data treatment that are often misinterpreted or not fully understood, such as model selection, ANOVA results, and residual analysis. We will focus on statistical data analysis for an optimization design, a face-centered central composite design to optimize the chromatographic conditions for the separation of three compounds, bisdemethoxycurcumin (BMC), demethoxycurcumin (DMC), and curcumin (CUR), in two different software: Design Expert and Fusion QbD. This data set was selected for a didactic purpose, and a CCD design was chosen since it is a common and efficient DOE method used for the optimization and generation of MODR. All the topics discussed in this paper can (and should) be applied to any chromatography separation employing the DOE within the AQbD framework.

## 2. Materials and Methods

### 2.1. Chemicals and Instrumentation

The solvents used for sample preparation and as a mobile phase were acetonitrile (Honeywell, Charlotte, NC, USA) and analytical-/chromatographic-grade ethanol (Honeywell, Offenbach am Main, Germany). The standard containing all three curcuminoids, bisdemethoxycurcumin (BMC), desmethoxycurcumin (DMC), and curcumin (CUR), was obtained from Neon Comercial Ltda. (Suzano, Brazil). All samples were filtered using 0.22 µm syringe filters before injection into the chromatographic system. The column was obtained from YMC Process Technologies YMC-Triart C18 (1.9 µm, 100 mm × 2.1 mm) (Devens, MA, USA). The chromatographic analyses were performed in an ACQUITY UPLC system with an automatic injector and binary pump (Waters Co., Milford, MA, USA). The detector used was a diode array detector (DAD) (Waters Co., Milford, MA, USA) with a wavelength range of 190 to 500 nm.

### 2.2. Curcuminoid Solutions

The curcuminoids were prepared at a concentration of 0.1 mg mL^−1^ in ethanol. A 100 µL aliquot was diluted from these solutions in 5 mL of an ethanol/water (50/50%) (*v*/*v*) solution.

### 2.3. Face-Centered Central Composite Design

A central composite design (CCD) was performed to optimize the chromatographic conditions for the separation of curcuminoids. The studied factors and their levels are shown in Table 1. The values of the factor levels were defined based on prior knowledge and screening studies.

The flow rate of the mobile phase, column oven temperature, and acetonitrile percentage (mobile phase composition) were the variables that most impacted the separation of the analytes; therefore, these parameters were selected. The α = 1 (face-centered) was used due to restrictions in the experimental conditions of flow rate and temperature. The injection volume was fixed at 2 µL, and the wavelength of the DAD detector was fixed at 420 nm. In order to show the importance of including the sample preparation error in the DOE, the same DOE was performed twice, one with only a solution for all runs and the other with an independent solution for each experimental design run, totaling 19 independent solutions.

### 2.4. Data Treatment

Empower (Waters Co., Milford, MA, USA) was the chromatography data system (CDS) used to record and process the chromatograms. For the design of experiments, Fusion QbD Version 10.1 (S-Matrix, Eureka, CA, USA) was used in the liquid chromatography method development mode (LC method development), which allows for the import and export of data directly with Empower. The data obtained from the design of experiments were also evaluated in Design Expert Version 13.0.5.0 (StatEase, Minneapolis, MN, USA) to explore and compare different tools for model evaluation and MODR generation.

## 3. Results and Discussion

### 3.1. Model Significance Evaluation 

As the experimental design chosen was the face-centered central composite, a total of 19 experiments were obtained, including eight cubic points, six axial points, and five central points, which were run in random order. The responses evaluated were retention factor (k), tailing factor, and the resolution of the critical pairs (Rs) of the three curcuminoids: BMC, DMC, and CUR (for a total of eight responses). These chromatographic parameters are directly affected by the experimental conditions and therefore are important in the development of liquid chromatographic methods. The experimental matrix with the measured response values is shown in Table S1 in the Supplementary Materials.

The DOE experimental data (Table S1) were then submitted to regression analysis for the construction of mathematical models that express the relationship between the factors (CMPs) and the responses (CMAs) in the Fusion QbD and Design Expert software. For each response monitored, a model was generated; therefore, this study provided eight models. The least squares method (LS) was the used approach in regression analysis for estimating regression coefficients (*β*). This method is widely employed for modeling data, aiming to determine coefficients by minimizing the sum of the squares of the residuals (*SSr*) [21]. The general solution for fitting a least squares model, in matrix notation, is given as follows:

(1) $$b={\left(X^{t}X\right)}^{-1}X^{t}y$$

In optimization design or response surface designs, polynomial models are most often built (2), which includes an intercept (*β*_0_), the main effects terms (*β_i_*), the interactions terms (*β_ij_*), and the quadratic terms (*β_ii_*) [6]. The complexity of the model depends on the design chosen and the number of factors studied. Therefore, a CCD can generate up to a second-order polynomial or quadratic model and the equation for k factors can be represented as follows:

(2) $$y=\beta_{0}+\underset{i}{\sum }\beta_{i}x_{i}+\underset{i}{\sum }\beta_{ii}x_{i}^{2}+\underset{i<j}{\sum }\underset{j}{\sum }\beta_{ij}x_{i}x_{j}+\varepsilon$$

where *y* represents the experimental response, *x_i_*, *…*, *x_j_*, are the factors studied, and *ε* is the random error.

However, usually, not all coefficients are statistically significant; the significant ones can be identified by applying model selection techniques, which will be described in the subsequent sections. Model fitting, model inference, and validation are critical procedures that must be carried out before using the model for interpretation and prediction. Regarding model inference, hypothesis tests may be used to evaluate the utility of the fitted model and the significance of the estimated parameters [22]. The first step in model inference is to assess the significance of the regression, i.e., to check whether the factors *x* influence *y*. To achieve this, a hypothesis test based on the F-distribution (F-test) is performed. The appropriate hypothesis for the overall test of model significance is as follows:

H_0_: *β*_1_ = *β*_2_ = … = *β_k_* = 0 (3)

H_a_: *β_i_ ≠* 0 for at least one *i* (4)

The null hypothesis (H_0_) (3) states that the regression coefficients are equal to zero, which means that a variation in *x* does not influence *y*. In this case, there is no model relating *y* to any *x* variable. On the other hand, the alternative hypothesis (H_a_) (4) assumes that at least one coefficient is different from zero, indicating that at least one factor has a significant effect on the evaluated response. After postulating the hypotheses, the F-test is used to compare two sources of variation provided by ANOVA, the mean square of the regression (*MS_R_*), and the mean square of the residuals (*MS_r_*) (5). These sources of the variation come from decomposing the sum of the total squares (*SS_T_*) into the sum of the squares of the regression (*SS_R_*) and the sum of the squares of the residuals (*SS_r_*), which can be written as *SS_T_* = *SS_R_* + *SS_r_*. These mean squares are obtained by dividing the sum of the squares by their respective degrees of freedom (*p* − 1) and (*n* − *p*) as shown in Equation (5).

(5) $$F_{0}=\frac{SS_{R}/\left(p-1\right)}{SS_{r}/\left(n-p\right)}=\frac{MS_{R}}{MS_{r}}$$

Here, *p* is the number of parameters in the model, and *n* is the number of experiments.

When the calculated F-value (*F*_0_) from Equation (5) exceeds the tabulated F-value (*F*_tab_) at the desired confidence level, the null hypothesis can be rejected, and the regression is significant. The higher the calculated F-value, the more significant the regression. Alternatively, the *p*-value can also be used for hypothesis testing, and H_0_ can be rejected if the *p*-value for the *F*_0_ is lower than the chosen significance level (α = 0.05). This means that the probability of the H_0_ being correct is lower than 5%. For example, based on the design with a single solution, the five-parameter BMC retention factor regression model is significant, as the F-value = 1825.11 is greater than the *F*_tab_ = 3.11 (F-distribution table) and the *p*-value < 0.05 at a 95% level of confidence (Table 2). The BMC retention factor model is shown in Equation (6). Consequently, the number of degrees of freedom for the model is four (Table 2), which is calculated as the number of parameters minus one (*p* − 1). For the residuals, the number of degrees of freedom is fourteen, as it is calculated as (*n* − *p*), the number of experiments (*n*) minus the number of parameters (*p*).

(6) $$k\;BMC=2.13-2.22\;\cdot \;B-0.518\;\cdot \;C+0.435\;\cdot \;BC+1.04\;\cdot \;B^{2}\;$$

After establishing the significance of the regression model through the F-test or *p*-value, the next step is to evaluate the explanatory power of the model, using the coefficient of multiple determination (*R*^2^) parameter as a metric. The *R*^2^ is used to assess the model’s overall adequacy, which shows how much of the variation related to the response can be explained by the coefficients (7).

(7) $$R^{2}=\frac{SS_{R}}{SS_{T}}=1-\frac{SS_{r}}{SS_{T}}$$

The closer the *R*^2^ values are to 1, the higher the amount of experimental variation of the response that is described by the model. However, a high *R*^2^ value does not always indicate that the regression model is adequate for its intended use. Adding a new coefficient to the model will increase the *R*^2^ value, regardless of whether it is statistically significant or not. Thus, the adjusted *R*^2^ (*R*^2^*_adj_*) and the predicted *R*^2^ (*R*^2^*_pred_*) can be used to obtain additional information on the explanatory power of the regression model [23]. The adjusted *R*^2^ indicates the percentage of variation explained by the significant coefficients (8) [24]; this means that the adjusted *R*^2^ only increases when the added coefficient improves the model and decreases if the added coefficient does not improve the model or if the improvement is lower than expected [23].

(8) $$R_{adj}^{2}=1-\frac{SS_{E}/\left(n-p\right)}{SS_{T}/\left(n-1\right)}=1-\left(\frac{n-1}{n-p}\right)\left(1-R^{2}\right)$$

where *p* is the number of parameters in the model.

The predicted *R*^2^ indicates the predictive capability of the regression model and can be computed using Equation (9), which involves the predicted residual sum of squares for the model (PRESS) (10).

(9) $$R_{Pred}^{2}=1-\left(\frac{PRESS}{SS_{r}+SS_{R}}\right)\;$$

(10) $$PRESS=\overset{n}{\underset{i=1}{\sum }}{\left(e_{-i}\right)}^{2}$$

Here, *e_-i_* is the residual calculated by fitting a model without the *i-th* run and then predicting the *i-th* observation using the resulting model.

(11) $$e_{-i}=y_{i}-{\hat{y}}_{i}=\frac{e_{i}}{1-h_{ii}}$$

As shown in Table 3, the models with the higher number of coefficients, including the non-significant ones, have the highest *R*^2^ values. However, the predicted *R*^2^ for the model with ten coefficients (0.9736) is lower than that for the model with five coefficients (0.9925), indicating the superior predictive capacity of the model with fewer coefficients. This aligns with the model obtained for the BMC retention factor, with five parameters. When choosing the most suitable model, it is important to consider the concept of parsimony, favoring simpler models with fewer coefficients over complex models when they fit the data similarly. As a result, models with high *R*^2^ values may produce inaccurate predictions of future observations or estimates of the mean response, i.e., smaller predicted *R*^2^ values due to model overfitting [23]. This happens because non-significant coefficients are actually describing random variation, which is not reproducible in future predictions. Ideally, a model should have both high *R*^2^ and high predicted *R*^2^, which suggests that the model not only explains the data well but also has the potential to make accurate predictions in future observations.

Furthermore, when the *R*^2^ and the adjusted *R*^2^ diverge more than 0.2, non-significant terms have likely been included in the model [25]. Thus, by considering both *R*^2^ and predicted *R*^2^, along with adjusted *R*^2^, a more complete evaluation of the regression model can be performed, ensuring that it not only explains the data well but also has the potential to make accurate predictions in future observations.

### 3.2. Model Selection

Using a model selection technique helps to evaluate and compare possible model coefficients. Several methods have been developed to assess small subsets of regression models by adding or removing a single coefficient at a time. These methods are generally called stepwise procedures and can be classified into forward selection, backward elimination, and stepwise regression [21].

Forward selection starts by including only the intercept as a coefficient in the model. The coefficients are included in the model one at a time until the optimal subset is found. The first coefficient to be selected for the model is the one with the largest simple correlation with the response, and it also provides the largest statistic to test the significance of the regression. The model will include the coefficient if the F-statistic is greater than the preselected F-value, also called *F*_IN_ or F to enter (F to add in Fusion QbD). The next coefficient chosen for inclusion in the model will be the one with the highest correlation after adjusting for the effect of the first coefficient added to the response. This procedure will continue until the partial F-statistic (12) at a certain step is smaller than the *F*_IN_ or until the last coefficient is added to the model [21,26].

(12) $$F-ratio=\frac{\left(SSR_{p}-SSR_{p-1}\right)/\left(p-1\right)}{\left(SSE_{p}\right)/\left(n-p\right)}$$

Here, *SSR_p_* is the sum of squares for the complete model, and *SSR_p_*_−1_ is the sum of squares for the model with the coefficients removed. This partial *F*-statistic (*F-ratio*) is similar to the *F*-value calculated in the regression significance phase. However, in this case, *MS_R_* is obtained from a partial sum of squares, and *MS_r_* comes from the full model with all parameters (*SSE_p_*). The terms (*p* − 1) and (*n* − *p*) are the degrees of freedom of the *MS_R_* and *MS_r_*, respectively.

Some software, such as Fusion QbD, can also report the t-statistic for adding or removing a variable because it can be considered a variation of the F-statistic, given that *t^2^*_α/2,υ_ = *F*_α,1,υ_ [26], where *α* is the significance level and *υ* is the degrees of freedom.

Conversely, backward elimination seeks to find the best-fitting model from the full model, including all K candidate factors. Then the partial statistical F is calculated for each regressor as if it were the last variable to enter the model, and the smallest F-value is compared to the preselected F_OUT_ (or F to remove in Fusion QbD). If the F-ratio < F_OUT_, the regressor is removed from the model. This procedure is then repeated for a regression model with K − 1 regressors. The backward elimination algorithm terminates when the smallest partial value *F-ratio* > *F*_OUT_. Stepwise regression is a modification of the forward selection procedure, where at every step, all the coefficients that entered the model are reevaluated using their partial F-tests or t-tests. Stepwise regression, therefore, requires two cut-off values, *F*_IN_ and *F*_OUT_. The choice of the *F*_IN/OUT_ cut-off value can be conceived as a stopping rule for the above procedures [27].

In Fusion QbD, data analysis can be performed through model regression (stepwise regression) with the options of backward elimination–design model and extended model, forward selection, or combined. All of these regression modes use the F to add/remove to select the model coefficients, with the default value of F to add/remove being equal to 4, which corresponds to a 5% in the *F*-distribution and is equivalent to a probability *p*-value of 0.05 used in a *t*-test [26].

Design Expert also uses model selection using stepwise regression in both forward and backward modes, which are performed in the same way as in Fusion QbD. However, the criteria used in Design Expert are the *p*-value and the adjusted *R*^2^. In addition, Design Expert provides options for selecting model terms based on information theory, demonstrating the relationship between likelihood and the amount of information lost when approximating the data with a model. Design Expert has two algorithms for model selection, including the corrected Akaike’s information criterion (*AICc*), which provides an estimate of the information lost when a specific model is used to represent the process that generated the data, i.e., the best model will be the one that approximates the data with the least loss of information and is best for small designs. Mathematically, the *AICc* is calculated using the following equation:

(13) $$AICc\;\left(M\right)=-2\ln\left(L\langle M|Data\rangle \right)+2p+\frac{2p\left(p+1\right)}{n-p-1}$$

where *p* is the number of terms in the model, including the intercept, random effects, and fixed effects, *n* is the number of lines in the plan, and the term (*L*[*M|Data*]) is the model fit measure (the larger this term is the better the fit).

The Bayesian information criterion (*BIC*) is another algorithm option in Design Expert and is similar to *AICc* but tends to favor models with fewer parameters. Equation (14) expresses *BIC* as follows:

(14) $$BIC=-2\ln(L\langle M|Data\rangle +\ln\left(n\right)p$$

The smaller *AICc* or *BIC* values represent a parsimonious model with a higher quality of fit. Thus, for model selection of the responses, the *p*-value (backward mode and α = 0.05) in Design Expert and the backward elimination–design model option (F = 4.0) in Fusion QbD were used as selection criteria. Table 4 shows that the selected model factors for BMC retention factor were quite similar for both software. However, since the Fusion QbD selection criterion is not based only on the *p*-value but considers that the *F*_0_ value must be greater than 4, the AC term was included in the model since it had an *F*_0_ = 4.069. This value is very close to 4, and considering the AC *p*-value = 0.0648, this coefficient should be removed from the model. A confidence interval (CI) at a 95% level of confidence can also be used to establish whether a coefficient is significant. If the CI passes through zero, that is, one of the limits is positive and the other is negative, the coefficient may be zero, indicating that the coefficient is not significant. As seen in Table 5, the confidence interval of the AC term includes zero and agrees with the *p*-value that this coefficient is not significant and should be removed from the model.

Another difference observed in Table 4 is that Fusion QbD does not include the term A, unlike Design Expert, which retains it to maintain the model hierarchy. Non-hierarchical models, like the one suggested by Fusion QbD, are advantageous in optimization-focused experiments because excluding non-significant terms reduces model variance, resulting in narrower confidence intervals for the mean response. On the other hand, the principle of hierarchy dictates that if an interaction term is included, its main effects must also be included, even if their *p*-values are not significant [28].

While hierarchical models retain non-significant terms, which can increase prediction variance, this often leads to wider confidence intervals, particularly when the inclusion of non-significant terms increases the residual mean square (*MS_r_*). In optimization studies, preserving hierarchy can yield an inferior model because it affects the empirical response surface and predicted values, both of which are critical for optimization. Therefore, when the primary goal is prediction, model parsimony becomes more important [21,29]. Since the AC interaction really is non-significant and will be removed from the model, this content will not be further developed. For more information on hierarchical and non-hierarchical models and their implications, see [21,28,29,30,31].

### 3.3. Lack of Fit Test

As mentioned before, the *R*^2^ does not guarantee that the model is adjusted to the data, which is a common misinterpretation. To assess whether the model fits the data well, a lack of fit F-test (15) is used to compare the variances of the lack of fit and the pure error, which ANOVA also provides. The lack of fit test relies on the estimation of pure error to compare it with the model error. If replicates are not available, it is not possible to estimate the pure error, making it difficult to determine if the model error is due to a lack of fit or just random variation. Usually, authentic replicates are performed in the central points of the DOE. Thus, the squared sum of the residuals is decomposed into pure error sum of squares (*SS_PE_*) and lack of fit sum of squares (*SS_LoF_*).

(15) $$F-Lack\;of\;Fit=\frac{SS_{LoF}/\left(m-p\right)}{SS_{PE}/\left(n-P\right)}=\frac{MS_{LoF}}{MS_{PE}}$$

Here, *MS_LOF_* is the mean square of lack of fit, and *MS_PE_* is the mean square of pure error.

In Table 2, the BMC retention factor model showed a lack of fit (F-value = 320.87 and *p*-value < 0.05). Although the test results indicate a lack of fit, additional considerations must be taken before concluding that the model is not appropriate; as suggested by Equation (15), the inverse relationship with the pure error highlights that a small pure error value can significantly amplify the *F*-value, increasing the likelihood of identifying a model with a lack of fit, when in fact, this is not true. This small pure error, however, raises concerns regarding the potential underestimation of experimental error, caused by the absence of authentic replicates during the execution of the DOE. In this DOE example, a single solution was used, resulting in the computation of solely the small variability intrinsic to the chromatographic equipment in the pure error term. Underestimating the pure error due to the lack of authentic replicates is a very common mistake made in a DOE study, and many examples can be found in the literature, in which the same values of responses were obtained (in this extreme case, the pure error equals zero, and therefore, it is not possible to carry out the lack of fit *F-*test).

To exemplify the effect of sample preparation, the same DOE was conducted again, this time using nineteen independent solutions—allocating one solution for each run—which ensures that the models encompass the maximum amount of variability (Table 5).

A regression analysis was performed on the data from the DOE using independent curcuminoid solutions, and the same models as in the DOE with a single solution were generated. The ANOVA results for the BMC retention factor in Table 6 show that the model is significant, with the adjusted and predicted *R^2^* values slightly higher and very close to the values found in the previous DOE. As expected, the pure error value increased, resulting in a much lower *F*-value in the lack of fit test (*F*-value = 8.97, Design Expert) and a *p*-value close to 0.05. Since the pure error was derived from the replicates at the central point, authentic replication at this point should be sufficient to provide a reliable estimate of the pure error, making the experimental work more practical. We have compared the results of entire replication and replication only at the center points, and the results were very similar. In this study, the mobile phase was mixed using the chromatograph (binary pump), and there was no pH adjustment, thereby minimizing variation from these experimental variables. Consequently, only sample preparation was considered in the preparation of the authentic replicates.

Even though the model’s lack of fit remains statistically significant at the 95% confidence level, this result might not have practical implications. Other parameters, such as residual analysis, should be checked to evaluate the quality of the model fit and determine whether the model can be employed for its intended purpose.

### 3.4. Residual Analysis and Diagnostic Plots

The *t*- and *F*-statistics, as well as *R^2^*, reflect global model properties, and the evaluation of only these parameters does not guarantee that the model is adequate. In general, examining the model fit is essential to ensure that it provides an acceptable approximation of the true system and that none of the least squares regression assumptions are violated [32].

Residual analysis is the basis of most diagnostic procedures to detect failures of basic assumptions of the regression model [7,21]. Normality, homoscedasticity, and independence are the major assumptions about the residuals that need to be verified. It is also useful for identifying concerning data points using leverage and influence point analysis. The residuals are defined as the difference between the observed (*y_i_*) and the estimated value by the model $\left(\hat{y_{i}}\right)$.

(16) $$e_{i}=y_{i}-{\hat{y}}_{i},\;i=1,\;2,\;3,\;n.$$

**Figure 1:**
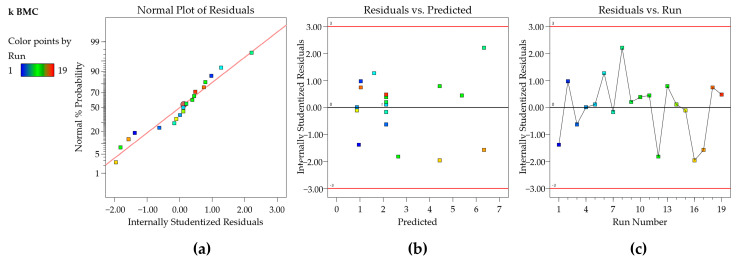
Diagnostic plots from Design Expert software—(**a**) normality plot; (**b**) residuals vs. predicted; (**c**) residuals vs. run. The red line in b and c represents the boundaries of ±3 Studentized residuals, which denotes a 99.7% confidence level. For a 95% confidence level, the points should be within the ±2 Studentized residual limits.

Thus, the residuals are a measure of the variability in the response variable that the regression model does not describe, and the analysis of the residual plots is also a key step in evaluating the model adequacy and is an effective way to determine how well the regression model is adjusted to the data [21]. To build these plots, scaled residuals (externally and internally Studentized residuals, standardized residuals, or residuals, ...) are used to aid in the visualization of points [33]. The *F*- and *t*-statistics, along with confidence and prediction intervals, all rely on the assumption that the residuals have a normal distribution. To check this assumption, a normal probability plot of the residuals is used to visualize if the residuals follow a straight line with emphasis on the central values, as seen in Figure 1a. Some scattering is expected, even for normal data (represented by a soft “S-shaped” curve), but the presence of defined patterns, such as a strongly well-defined S-shaped curve, suggests that the response needs to be transformed to improve the model fit [33].

The plot of residuals as a function of the predicted response values is used to test the assumption of constant variance (homoscedasticity). The plot should present a random scatter, which means a constant range of the residuals across the graph (Figure 1b) [33]. If the scatter expands following a “megaphone pattern”, this suggests that the data may need a transformation or the range of experimental design should be decreased. The independence (uncorrelation) of the residuals can be verified using the plot of the residuals as a function of the experiment run order. This plot should present a random pattern, indicating that there is no influence of an external factor or an error during the execution of the experiment (Figure 1c) [33]. Also, in Figure 1b,c, the points should be within the limits ± 2 of Studentized residuals (residuals/regression standard deviation) for a 95% confidence level (Fusion QbD default) and ±3 Studentized residuals for a 99.7% confidence level 99.7% (Design Expert default). Points outside or near these boundaries should be checked as possible outliers (transcription errors should be verified and a new experiment carried out, if possible). The external standardized residuals present a similar metric; however, the residuals are calculated using a model in which the point was not included [34]; therefore, they are more sensitive to outliers.

**Figure 2:**
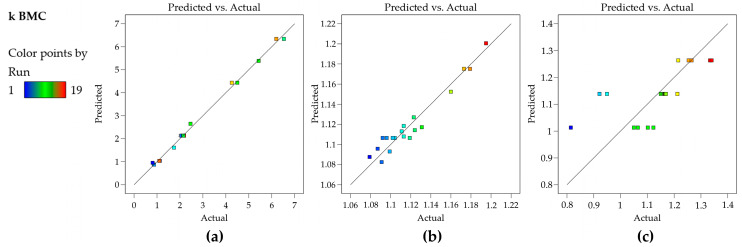
Predicted vs. actual plots from Design Expert: (**a**) a good model fit (data from k BMC model of this study); (**b**) good predictive capability but high experimental error; and (**c**) inadequate model. (**b**,**c**) Graphs from other data sets of our group.

The residual plots have shown normality, homoscedasticity, and independence, indicating that the model is appropriate to describe the experimental responses obtained. Finally, the plot of observed versus predicted values is very useful as a diagnostic for model prediction capability [35]. In Figure 2a, the plot shows that the residuals are close to the straight line, which means that the predicted values are in good agreement with the experimental values; therefore, the model for the k BMC can be used for prediction. Figure 2b,c bring graphs for other data sets for an illustrative purpose. Figure 2b shows residuals of a model that presents *R*^2^ of 0.938 and still presents a good predictive capability (there is no indication of lack of fit, but rather the experimental error is high and therefore cannot be described by the model, which leaves higher residuals than the ones shown in Figure 2a). Figure 2c shows an inadequate model (*R*^2^ of 0.48) and a poor prediction capability, showing that for a broad range of the experimental data, the predicted value is the same; therefore, the corresponding model cannot be used either for the prediction or construction of Design Space/MODR.

Other residual graphs that should be analyzed are influential plots, such as Leverage and Cook’s distance, due to their ability to detect influential points—data points that significantly impact the regression coefficients and predictions. Even though influential points significantly impact the regression results, they are not necessarily outliers, and they can be identified by assessing both leverage and residual graphs. Leverage measures how far an individual predictor value deviates from the mean of the predictor values; points with high leverage have a greater potential to affect the regression model’s estimates [21,36]. For instance, a single influential point with high leverage and a large residual can alter the slope and intercept of the regression line, leading to misleading conclusions. Leverage is calculated using the hat matrix, denoted as *H*. For a given data point *i,* leverage *h_i_* is derived from the diagonal elements of this matrix (17) [21,34].

(17) $$Leverage\;\left(h_{i}\right)=diag\left(H\right)$$

Specifically, *H* is obtained as follows:

(18) $$H=X{\left(X^{T}X\right)}^{-1}X^{T}$$

where *X* is the design matrix, with a row for each execution in the project (n) and a column for each term in the model (p).

Leverage values range from 0 to 1, with higher values indicating that the corresponding data point has a greater potential to influence the regression model’s parameter estimates. Points with leverage substantially larger than the average leverage, ($\frac{p+1}{n}$), are considered high leverage points and require closer examination [34,36]. Figure 2b shows that no point in the k BMC model has high leverage since all points are located below the calculated average leverage value (red line).

Cook’s distance (*D_i_*), which is described by Equation (19), is another important diagnostic that combines the information from leverage and residuals to quantify the influence of each data point on the fitted regression model [36]. Cook’s distance is a summary of how much a regression model changes when the *i*th observation is removed.

(19) $$D_{i}=\frac{r_{i}^{2}}{p+1}\;\left(\frac{Leverage_{i}}{1-Leverage_{i}}\right)$$

**Figure 3:**
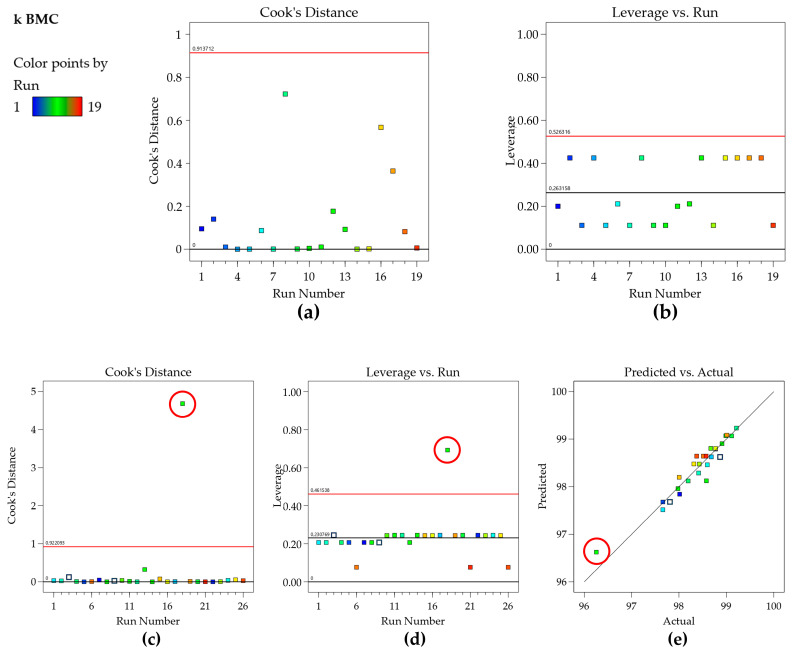
(**a**) Cook’s distance and (**b**) leverage plots for the k BMC model; (**c**) Cook’s distance for another data set, indicating a point above the threshold; (**d**) leverage plot highlighting the same point; and (**e**) predicted vs. actual graph indicating that the point is well predicted by the model.

Cook’s distance plot visually represents this influence, where points with a large Cook’s distance value (typically greater than 1) are considered influential and may have a negative impact on the model’s predictions [34]. Evaluating Cook’s distance graph for the retention factor of BMC (Figure 3a) confirms this, as no point exceeds the calculated threshold. Therefore, no outlier or influential point is present in the data set. Identifying such points is crucial for ensuring the robustness and reliability of the regression model, as influential points can distort the model’s outcomes and lead to misleading conclusions.

Attention should be paid so as not to exclude a given point with a high Cook’s distance (circled in red) by interpreting it as an outlier when it is actually a high leverage point that is well described by the model, as shown in Figure 3c–e (another data set of our group). Another metric sometimes used is DFFITS, and although the raw values resulting from the equations are different, Cook’s distance and DFFITS are conceptually identical, and there is a closed-form formula to convert one value to the other [34,36].

All eight models, Equation (3) and Equations (20)–(26), obtained in this study went through all the evaluation steps described so far. The ANOVA tables, fit statistics, and residual plots for these models are available in the Supplementary Materials.

(20) $$k\;DMC=2.43-2.51\;\cdot \;B-0.522\;\cdot \;C+0.435\;\cdot \;BC+1.18\;\cdot \;B^{2}$$

(21) $$k\;CUR=2.76-2.85\;\cdot \;B-0.515\;\cdot \;C+0.428\;\cdot BC+1.34\;\cdot \;B^{2}$$

(22) Tailing BMC=1.12−0.021· A+0.048 · B−0.018 · AB

(23) Tailing DMC=1.12−0.019 · A+0.039 · B−0.014 · AB

(24) $$Tailing\;CUR=1.11-0.013\;\cdot \;A+0.030\;\cdot \;B+0.011\;\cdot \;C+0.013\;\cdot \;BC+0.031\;\cdot \;B^{2}$$

(25) $$Rs\left(BMC,DMC\right)=2,91-0.039\;\cdot \;A-0.788\;\cdot B+0.372\;\cdot C+0.050\;\cdot \;AC-0.212\;\cdot \;BC-0.088\;\cdot \;C^{2}$$

(26) $$Rs\left(DMC,\;CUR\right)=3.01-0.036\;\cdot \;A-0.727\;\cdot \;B+0.367\;\cdot \;C+0.041\;\cdot \;AC-0.194\;\cdot \;BC-0.073\;\cdot \;B^{2}+-0.065\;\cdot \;C^{2}\;$$

### 3.5. Response Surface

**Figure 4:**
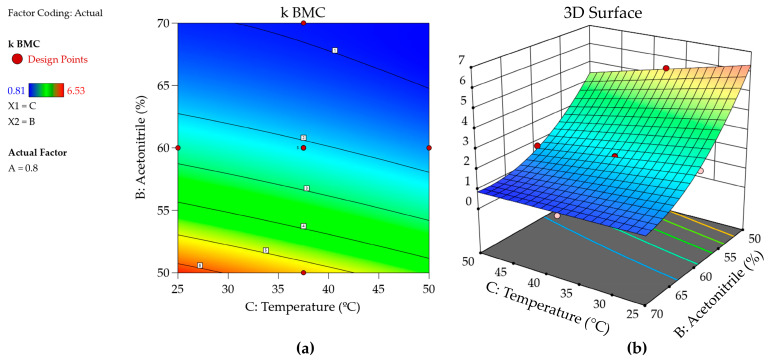
(**a**) Counter plot and (**b**) the 3D mean response surface of the k BMC response.

In the optimization step, once the model is considered suitable for its intended use, it is possible to use response surfaces or contour plots to help the interpretation and find the optimal location point or region [10]; both graphs are available in Design Expert and Fusion QbD. These graphics are an illustration of the built model and describe the behavior of the response measured concerning the range of factors assessed and the fitted model. Therefore, assessing the most significant coefficients of each model is important. In Figure 4, it can be observed that the retention factor increases as the percentage of acetonitrile decreases. This observation aligns with the principle of reversed phase chromatography that lowering the percentage of the strong elution solvent increases retention.

Furthermore, using multivariate methods such as DOE enables the identification of interactions between the factors studied. Figure 4 illustrates that temperature does not significantly influence the retention of the BMC compound in 70% acetonitrile. Conversely, when the percentage of acetonitrile is reduced, the temperature begins to affect retention; specifically, lower temperatures result in a higher retention of the compounds. This indicates an interaction between temperature and the percentage of acetonitrile. Graphically, these interactions are characterized by non-parallel lines on the response surface, further demonstrating the presence of factor interactions. However, the response surfaces only represent a region in which the responses are observed on average and therefore give no guarantee that the responses will meet the predefined criteria with high probability [8,10].

### 3.6. MODR Construction

**Figure 5:**
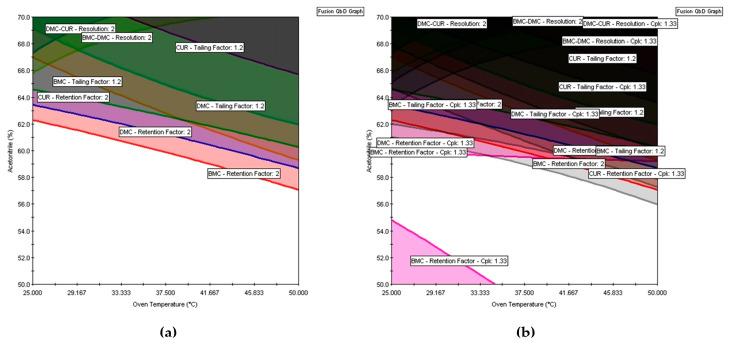

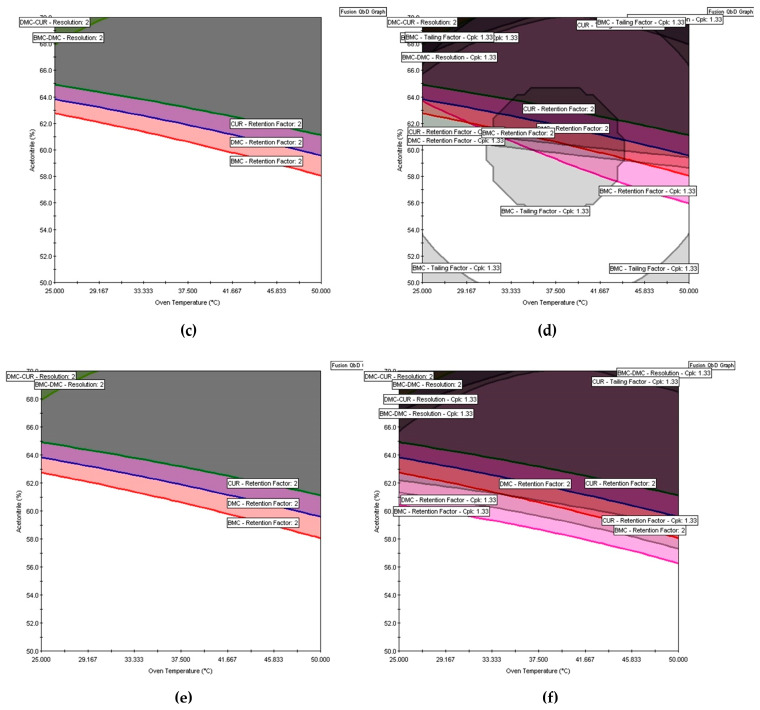
Contour maps (**a**) and MODR (**b**) calculated using the capability index Cpk, both constructed from the DOE with a single solution. Contour maps (**c**) and MODR (**d**) from the DOE with the independent solutions and using F to remove = 4 for model selection. Contour maps (**e**) and MODR (**f**) from the DOE with the independent solutions and using F to remove = 5 for model selection. The color lines represent areas of non-acceptable mean performance for each response: BMC retention factor (pink and orange); DMC retention factor (blue); CUR retention factor (green); BMC-DMC resolution (yellow); DMC-CUR resolution (dark green). The color grey was used to represent the *C_pk_* for all responses evaluated.

From DOE-derived models, knowledge is acquired, allowing access to a region where the behavior of analytical responses and method performance can be understood based on variations in the analytical parameters [8]. This region, known as the knowledge space, is confined to the experimental domain in the context of empirical models. In Figure 4 and Figure 5, the knowledge space is represented by the entire region of the graph. Within the knowledge space, areas where analytical parameters and their ranges fail to meet specifications, termed as the “non-acceptable performance region”, and areas where mean responses meet specifications, referred to as the “acceptable mean performance region”, can be identified [9].

Multiple response optimization tools are used to find the acceptable mean performance region when several responses are studied simultaneously. There are two primary methods for multiple response optimization. The first is graphical optimization, often referred to as overlay graphs, in which the contour plots of each response model are superimposed to estimate the region of intersection, thereby providing a region of optimal conditions or an acceptable performance region. The second method is based on the desirability functions (often called “numeric optimization” or Derringer and Suich desirability) [10]. Initially, individual desirability functions, di(*ŷ_i_*), for each *ŷ_i_* must be created using fitted models and establishing the optimization criteria. Desirability values always range from 0 to 1, with desirability equal to zero representing an undesired response and di(*ŷ_i_*) = 1 for the optimal response. Desirability functions can be customized to suit the chosen optimization criteria. These criteria typically fall into the following categories:

Maximize: The objective is to elevate the response variable to its maximum potential. Desirability increases as the response approaches its highest value.

Minimize: This criterion minimizes the response variable, striving for the lowest achievable value. Desirability increases as the response approaches its minimum value.

Target: The focus is on achieving a specific target value for the response variable. Desirability decreases as the response deviates from this target, emphasizing alignment with the desired outcome.

In range: which specifies a range (lower limit–upper limit) for acceptable results.

Cpk: the process capability index, which calculates the number of standard errors of the predicted response that are within the specification limits.

By tailoring desirability functions according to these criteria, the optimization process can effectively pursue the desired objectives for the analytical method. Once all n variables are transformed into desirability functions, they are combined into a single function called overall desirability (D), which is employed to discover the best combinations of responses. In mathematical terms, the overall desirability is given by the geometric mean of the n individual desirability shown by Equation (27) [10].

(27) $$D=\sqrt[{n}]{d_{1}d_{2\ldots }d_{n}}$$

In Design Expert, graphical and numerical (desirability) optimizations are available, whereas Fusion QbD uses only desirability to find the best overall answer. In Design Expert, the graphical optimization criteria are defined by the values specified by the analyst for the lower and upper limits, or sometimes just one of these limits.

**Figure 6:**
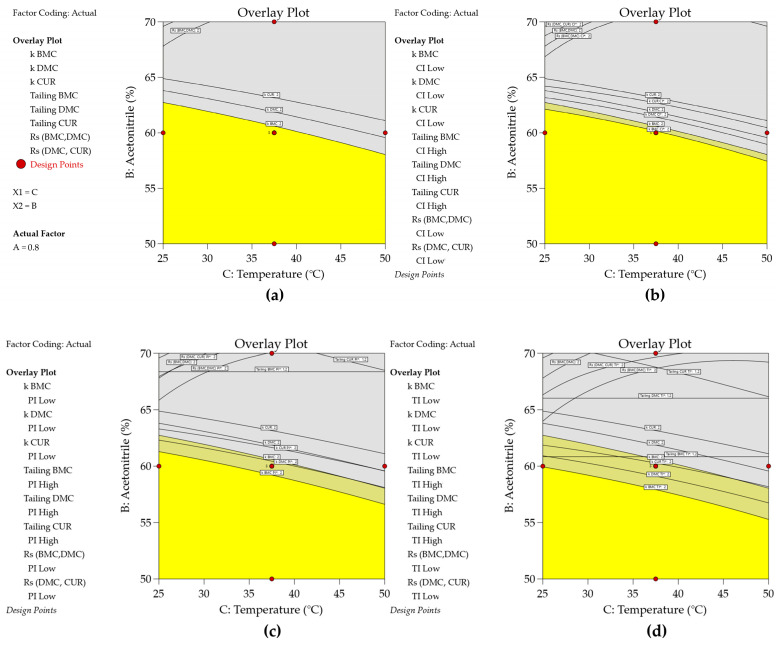
Overlay plots obtained using graphical optimization in Design Expert (**a**) with a confidence interval of α = 0.05 (or at 95% confidence level) (**b**), a prediction interval of α = 0.05 (**c**), and a tolerance interval of α = 0.05 and β = 0.99 (**d**). This optimization process was executed with the models from the DOE with the authentic replicates (the same data used to generate the MODR in Figure 5f).

All eight models (3, 20–26) were used to find the acceptable mean performance region. In Fusion QbD, the responses k BMC, k DMC, and k CUR were maximized with a lower bond of two in order to provide adequate retention. To ensure that the peaks were symmetrical, the tailing BMC, tailing DMC, and tailing CUR responses were minimized with the upper bound equal to 1.2. To ensure that adjacent peaks have adequate separation and avoid coelution, the response Rs (BMC, DMC) and Rs (DMC, CUR) were maximized with a lower bound equal to two. The same criteria were adopted for graphical optimization in Design Expert. The results of the multiple response optimization are shown in Figure 5a and Figure 6a.

In Figure 5a of Fusion QbD, the white regions indicate areas of acceptable mean performance, while the colored regions denote areas of non-acceptable mean performance. Similarly, in Design Expert’s graphical optimization shown in Figure 6a, the bright yellow regions imply acceptable mean performance, and the gray regions indicate unacceptable performance. However, like the mean response surfaces, these regions only represent the average performance and do not guarantee that the method will meet the requirements of its intended use. Furthermore, these regions cannot be referred to MODR because, according to GC USP <1220>, MODR is defined as a “multivariate space of analytical procedure parameters that ensure the ATP is fulfilled and therefore provide assurance of the quality of the measured value” [3]. Moreover, the MODR should ensure quality, which implies that it should represent a robust region as described in ICH Q14 [4]. Therefore, the design of MODR should incorporate the method uncertainties to determine whether the analytical procedure will assure quality. If a method is robust “on average” but exhibits excessive variation, it may fail to meet its robustness criteria [26]. Robustness incorporation includes estimating the variability of analytical measurements related to the CMAs. Also, each source of error is identified to increase analytical performance by reducing the variability associated with the analytical parameters.

Fusion QbD uses Monte Carlo simulations to include the measurement uncertainty of model parameters and estimate the probability of meeting CMA specifications. To start the simulations, the maximum expected variation (±3σ value) of each CMP must be entered. Then, the robustness indices should be selected, and the lower and upper specification limits (*LSL* and *ULS*, respectively) (26) should be defined for each CMA. These specification limits represent the MODR boundaries for each response and are also known as edges of failure. In Fusion QbD, process capability indices, such as *C_p_*, *C_pk_*, *C_pm_*, and *C_pkm_*, are implemented as robustness capability metrics to quantify system robustness [26]. These are standard statistical process control (SPC) metrics widely used to quantify and evaluate method variation in critical quality and performance characteristics. The *C_p_* index measures the potential capability of a process and is used when the response has a specified maximum allowable variation and symmetrical upper and lower specification limits (*USL* and *LSL*). It can be calculated as follows:

(28) $${\hat{C}}_{p}=\frac{USL-LSL}{6\sigma }$$

where 6*σ* represents the inherent variation in the average response value, constrained within the limits of a 3*σ* confidence interval. The *C_pk_* index measures the actual capability of a process, accounting for how centered the process is between the specification limits. If the goal of the response is to maximize, only the *LSL* is established (29). If the objective is to minimize, then only the *USL* is established (30).

(29) $${\hat{C}}_{p,\;lower}=\frac{\bar{x}-LSL}{3\sigma }\;$$

(30) $${\hat{C}}_{p,\;upper}=\frac{\bar{x}-USL}{3\sigma }\;$$

The *C_pm_* index is used when the response has a target value, and the specification limits are symmetrical (31). When the specification limits are asymmetrical, the *C_pkm_* index is used (32).

(31) $${\hat{C}}_{pm}=\frac{{\hat{C}}_{pk}}{6\sqrt{s^{2}+\left(\bar{x}-T\right)}}\;$$

(32) $${\hat{C}}_{pkm}=\frac{{\hat{C}}_{pk}}{\sqrt{1+\left(\frac{\bar{x}-T}{s}\right)}}$$

When *C_p_* = 1.00, it indicates that the process variation exactly matches the specification limits, meaning that the process spread (±3*σ*) is equal to the distance between the upper and lower specification limits (*USL* and *LSL*). This implies that approximately 99.7% of the process output falls within the specified limits, but it also suggests there is no room for error or drift in the process mean. When *C_p_* = 1.33, the specification limits are four standard deviations (±4*σ*) away from the process mean, compared to three standard deviations in the case of *C_p_* = 1.00. This allows for greater assurance that the process outputs will remain within the specified limits, even if there are minor variations or shifts in the process. This value suggests that approximately 99.99% of the process output will meet the specified limits. When *C_p_* = 2.00, it reflects an exceptionally capable process, where the process spread is only half of the specification range. This indicates that almost all the output will be within the specification limits, demonstrating a highly robust and reliable process with substantial tolerance for variations in process parameters [37]. Higher *C_p_* values, therefore, indicate better process capability and a reduced risk of producing out-of-specification products. Subsequently, the Monte Carlo simulation method begins by modeling the system, including input factors CMAs and their variability, characterized by probability distributions. The simulation generates numerous sets of input values through random sampling, each representing a possible scenario. The model calculates the corresponding analytical response for each set, repeating this process thousands to millions of times to produce a distribution of outcomes. This distribution reflects the combined measurement uncertainty of the analytical response, accounting for the variability of all input factors. By analyzing the outcomes, Monte Carlo simulations estimate the likelihood that the analytical method meets specified performance criteria under different conditions.

The desired levels of robustness for each study factor computed for the MU estimation were inserted as follows: (A) flow rate ± 0.024 mL/min, (B) % of organic solvent in the mobile phase ±1.5%, and (C) column temperature ± 1.5 °C. The *C_pk_* was the robustness index chosen for all responses. For the k BMC, k DMC, and k CUR responses, the goal was to maximize, and the *C_p, lower_* was calculated by setting up the *LSL* at 2.00 so the compounds have a minimal retention factor of two. To obtain symmetrical peaks for all compounds, the tailing responses were minimized, and the *C_p, upper_* was calculated setting the *ULS* of 1.2. For adequate separation of the critical pairs, the Rs (BMC, DMC) and Rs (DMC, CUR) responses were maximized, and *C_p, lower_* was calculated by setting up the *LSL* at 2.00. In this study, the risk control strategy involved selecting conditions that achieve *C_pk_* ≥ 1.33 for all the responses.

Figure 5b,d,f shows that after the robustness assessment using Monte Carlo simulations and the insertion of uncertainties into the MODR, the white region is smaller than that obtained without the robustness assessment. It is also possible to see how authentic replicates impact the MODR by comparing Figure 5a,b (results of the DOE without authentic replicates) to Figure 5e,f (results of the DOE with authentic replicates for all the runs). The importance of selecting the models that will be used to build the MODR can also be demonstrated. In Figure 5c,d, for example, the plots were constructed based on the DOE with authentic replicates using the value of *F* = 4, which resulted in the inclusion of a non-significant term in the tailing BMC model, as explained in the model selection section. When removing this term by increasing the *F-*value to 5, the MODR changes again (Figure 5e,f).

On the other hand, Design Expert uses the propagation of error (POE) method to identify factor settings that minimize the variation transmitted to a response from factors that are not fully controllable, thereby making the process more robust to input variations. This approach involves applying partial derivatives to locate flat areas on the response surface, which are less sensitive to variations. POE methods require a second-order hierarchical response surface model and estimated standard deviations for the numeric factors. These standard deviations can be inputted for all factors on the column info sheet or individually by selecting specific columns or cells and adjusting them in the design properties tool. The same uncertainty values used in Fusion QbD were applied in Design Expert. Using this information, the software constructs a response surface map that shows how factor variations affect each response. By employing multiple response optimization, including objectives to minimize the POE, optimal factor settings can be determined to achieve desired response goals with minimal variation [11]. The robustness assessment in Design Expert utilizes interval estimation to quantify variability and uncertainty in CMAs and CMPs. Interval estimates, including confidence, prediction, and tolerance intervals, can be added to graphical optimization plots (Figure 6) by selecting the “Show Interval” box and specifying the type and confidence level [38]. Unlike confidence intervals (CI) [39], which quantify the uncertainty of an estimated population variable (such as the mean or standard deviation), prediction and tolerance intervals address the uncertainty of future observations. A CI provides a range that is likely to contain the unknown population value, indicating the precision of a sample estimate (33). Therefore, it is worth emphasizing that the use of CI is not recommended for evaluating robustness.

(33) $$\hat{y}\pm \;t_{\left(1-\frac{\alpha }{2},\;n-p\right)}\cdot SE$$

(34) $$SE=s\cdot \sqrt{x_{0}{\left(X^{T}X\right)}^{-1}x_{0}^{T}}$$

Here, *SE* is the standard error of the design at *x*_0_, and *x*_0_ is the expanded point vector. In a predictive model, the expanded point vector is a list of values and their interactions and is a way to represent the settings of the factors for a particular location for purposes of prediction. It can be seen as a single-row matrix, with one element for every term in the model; it resembles a row of the expanded model matrix (*X*). The elements of the point vector and the terms represented by the model matrix’s columns must be in the same order. At last, *s* is the estimated standard deviation.

In contrast, a prediction interval (PI) [39] accounts for both the uncertainty in estimating the population mean and the random variation of individual values, thus offering a range within which the average of a future sample is expected to fall (35).

(35) $$\hat{y}\pm \;t_{\left(1-\frac{\alpha }{2},\;n-p\right)}\cdot SE_{pred}$$

(36) $$SE_{pred}=s\cdot \sqrt{1+x_{0}{\left(X^{T}X\right)}^{-1}x_{0}^{T}}$$

Here, *SE_pred_* is the predicted error for one future response measurement at *x*_0_.

Consequently, PIs are always wider than CIs (Figure 6b,c). Tolerance intervals (TIs) [39] are even broader, as they include a specified proportion of all individual outcomes, accounting for observed and unobserved variability within the population (Figure 6d). In mathematical terms, the *TI* is represented as follows:

(37) $$\hat{y}\pm \;s\cdot \left(TI\;Multiplier\right)$$

(38) $$TI\;Multiplier=t_{\left(1-\frac{\alpha }{2},\;n-p\right)}\cdot \sqrt{x_{0}{\left(X^{T}X\right)}^{-1}x_{0}^{T}}+\phi^{-1}\left(0.5+P/2\right)\cdot \sqrt{\frac{n-p}{X_{\left(\alpha ,\;n-p\right)}^{2}}}$$

where *p* is the number of terms in the model, *P* the proportion of the population contained in the tolerance interval, *ϕ* is the inverse normal function to convert the proportion to a normal score, and *X*^2^ is the chi-squared critical value [39].

Estimating prediction and tolerance intervals is crucial for evaluating the total analytical error (TAE) and assessing robustness in the analytical quality by design (AQbD) framework. For robustness studies and procedure qualification in the AQbD framework, determining the range in which future response values are likely to be found is critical for proper risk assessment. Using wider prediction and tolerance intervals can more accurately resolve this issue. In contrast, confidence intervals are less suitable for quantifying uncertainty in future observations and may not adequately support the design of the MODR [9]. By incorporating interval estimates, the graphical optimization (Figure 5) becomes a more powerful tool for visualizing and managing the uncertainty associated with process optimization, thereby enhancing decision-making and process robustness. As shown in Figure 6, the pale yellow color represents the region where the point estimate fulfills the criteria requirements but a portion of its interval estimate does not. This region becomes wider when moving from CI (Figure 6b) to PI (Figure 6c) and TI (Figure 6d).

**Figure 7:**
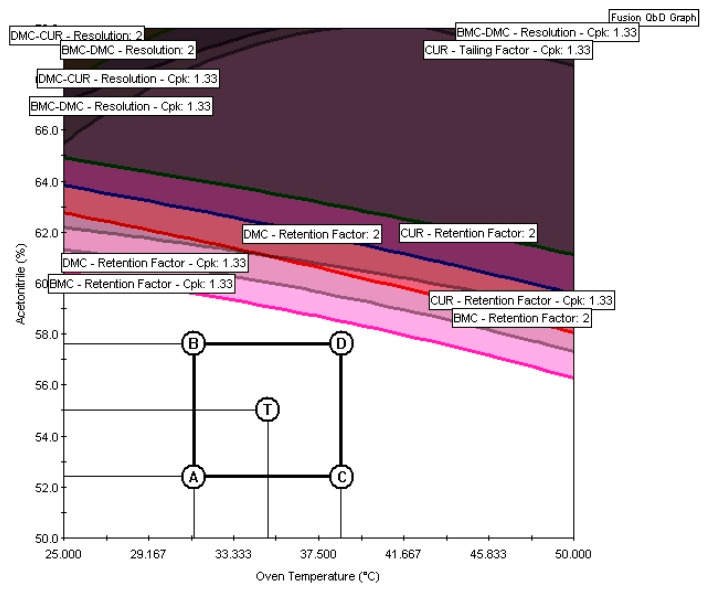
MODR with the PARs from Fusion QbD. The points A, B, C, D, and T represents the experimental conditions where the verification runs were performed. The experimental results and predicted results can be seen in Table S10 of the Supplementary Material.

Despite the differences in the software calculations and insertion of uncertainties, the regions delimited as MODR in Design Expert (Figure 6d) and Fusion QbD (Figure 5f) were the same. Once the MODR has been established, verification runs can be carried out to evaluate performance and provide additional evidence that the MODR is valid. Since the MODR is an irregular and multidimensional space, validating or qualifying the entire MODR can be challenging. Therefore, selecting a portion of the MODR for the verification runs is often practical. Fusion QbD verification runs can be performed by inserting the proven acceptable range (PARs), a rectangle added to the overlay graph within the MODR [26], as shown in Figure 7.

The conditions chosen for method validation were a flow rate of 0.825 mL min^−1^, a temperature of 35 °C, and 55% acetonitrile in the mobile phase, represented by point T in Figure 7. To check the method’s performance, the predicted values of the evaluated responses were compared with the experimental values (Table 7).

The Fusion QbD provides the predicted value and a corresponding lower and upper limit of the two-sigma confidence interval. According to Table 7, all the experimental results were close to the predicted value and within the two-sigma confidence interval, ensuring that under these conditions the method met the quality performance requirements.

It is important to note that if verification runs were conducted after a prolonged time of column use of the column, the experimental values may deviate from the predicted values due to column degradation and a loss of separation efficiency. This is illustrated in Supplementary Material Table S10, in which verification runs were performed after a prolonged time of column use. While the retention factor values remained close to the predicted values and within the confidence interval, the tailing factor values were higher than the predicted, and the resolution values were lower. This indicates a decrease in the column’s efficiency. In this situation, the experimental results must be compared to the acceptance criteria of the CMAs. In this case, the acceptance criteria for the resolution between the critical pairs are met since the resolution values in Table S10 are greater than two at all points. However, the experimental results for tailing are at or slightly above the acceptance criterion of tailing, which is less than 1.2. Although the tailing values are slightly above the acceptance criterion, the resolution values above two guarantee the separation of the compounds.

## 4. Conclusions

The results of this study demonstrated the importance of a careful model evaluation, including ANOVA and residual analysis, to avoid incorrect interpretations and flawed decisions about model usability. An illustration of this was the BMC retention factor model, which had normal, homoscedastic, and independent residuals, even though the *F*-test indicated that it was not well-fitted. None of the diagnostic graphs for influential points suggested any issues with the model. Furthermore, the adjusted *R*^2^ provided a high level of data explanation, accounting for 99.59% of the variation, while the predicted *R*^2^ and the predicted versus actual values plot confirmed the model’s predictive capability.

This study also highlights the importance of model selection for MODR generation since the addition of non-significant terms includes random variability, creating a non-realistic MODR. Additionally, including uncertainties in the contour plots is crucial, either as prediction/tolerance intervals or Monte Carlo simulations. Overall, the careful analysis of all parameters and their practical importance is crucial for making reliable decisions based on the models. Finally, understanding the particularities of each software outcome is important for proper data analysis. Despite the differences in the Design Expert and Fusion QbD, such as model selection and multiple response optimization, the MODRs resulting from the two software were similar. It should be highlighted that the recommendations provided in this paper can be used to guide data treatment in any chromatography separation employing the DOE within the AQbD framework.

## Figures and Tables

**Table 1:** Factors studied in the face-centered central composite experimental design to optimize the chromatograph condition.

	Factors	Units	−α	−1	0	+1	+α
A	Flow Rate	mL min^−1^	0.6	0.6	0.8	1.0	1.0
B	Acetonitrile	%	50	50	60	70	70
C	Temperature	°C	25	25	37.5	50	50

**Table 2:** ANOVA results from Design Expert and Fusion QbD for the retention factor of BMC, using the data performed with a single solution of the curcuminoids for all runs.

Source	Design Expert					Fusion QbD				
	SS ¹	df ^2^	MS ^3^	F-Value	*p*-Value	SS ¹	df ^2^	MS ^3^	F-Value	*p*-Value
Model	50.73	4	12.68	1825.11	<0.0001	50.76	4	12.68	1825.90	<0.0001
Residual	0.0973	14	0.0069			0.0973	14	0.0069		
Lack of Fit	0.0972	10	0.0097	323.87	<0.0001	0.0972	10	0.0097	670.95	<0.0001
Pure Error	0.0001	4	0.0000			<0.0001	4	<0.0001		
Cor Total	50.82	18				50.85	18			

^1^ Sum of squares; ^2^ degrees of freedom; ^3^ Mean square.

**Table 3:** Comparison of *R*^2^, adjusted-*R*^2^, and predicted-*R*^2^ values when non-significant coefficients are removed from the BMC retention factor model.

Fit Statistic	Number of Coefficients					
	10	9	8	7	6	5
*R* ^2^	0.9977	0.9977	0.9977	0.9976	0.9969	0.9968
Ajusted-*R*^2^	0.9955	0.9959	0.9963	0.9965	0.9957	0.9959
Predicted *R*^2^	0.9736	0.9771	0.9893	0.9901	0.9902	0.9925

**Table 4:** Coefficients of the BMC retention factor model from Design Expert and Fusion QbD.

	Design Expert					Fusion QbD				
Factor	Coefficient Estimate	Standard Error	95% CI Low	95% CI High	*p*-Value	Coefficient Estimate	Standard Error	95% CI Low	95% CI High	*p*-Value
Intercept	2.13	0.0357	2.05	2.21	<0.001	2.127	-	2.05	2.2	
A	−0.016	0.0339	−0.09	0.0579	0.646	-	-	-	-	-
B	−2.22	0.0339	−2.29	−2.14	<0.001	−2.21	0.033	−2.87	−2.140	<0.001
C	−0.518	0.0339	−0.592	−0.4441	<0.001	−0.5183	0.33	−0.5896	−0.447	<0.001
**AC**	**0.075**	**0.0379**	**−0.008**	**0.1576**	**0.071**	**0.0745**	**0.0369**	**−0.0053**	**0.1542**	**0.648**
BC	0.435	0.0379	0.3524	0.5176	<0.001	0.436	0.0369	0.3563	0.5157	<0.001
B^2^	1.04	0.0493	0.9329	1.15	<0.001	1.04	0.048	0.9364	1.14	<0.001

**Table 5:** Experimental matrix of the face-centered central composite experimental design, with the responses studied using an independent curcuminoid solution for each run.

	Factor			Response							
Run	A	B	C	k BMC	k DMC	k CUR	Tailing BMC	Tailing DMC	Tailing CUR	Rs (BMC, DMC)	Rs (DMC, CUR)
1	0.6	50	25	6.53	7.27	8.08	1.053	1.093	1.113	3.131	3.133
2	0.8	50	37.5	6.20	6.93	7.74	1.058	1.086	1.099	2.872	2.922
3	0.6	70	50	1.12	1.27	1.43	1.193	1.186	1.160	1.925	2.035
4	1	60	37.5	1.10	1.25	1.41	1.148	1.141	1.123	1.823	1.935
5	0.8	60	37.5	2.46	2.76	3.09	1.096	1.092	11.087	2.498	2.581
6	0.8	60	37.5	2.17	2.48	2.82	1.126	1.134	1.113	2.980	3.066
7	0.8	60	37.5	2.11	2.41	2.74	1.111	1.112	1.091	2.904	3.001
8	0.6	70	25	5.43	6.20	7.06	1.087	1.093	1.124	3.723	3.713
9	1	50	50	0.81	0.94	1.09	1.121	1.139	1.179	2.049	2.164
10	0.8	60	50	2.18	2.48	2.83	1.119	1.122	1.104	2.924	3.005
11	1	50	25	2.14	2.44	2.77	1.115	1.118	1.096	2.902	2.990
12	1	70	25	2.06	2.35	2.68	1.138	1.139	1.119	2.880	2.996
13	0.8	70	37.5	2.14	2.44	2.78	1.102	1.117	1.092	2.906	2.997
14	0.8	60	25	2.15	2.46	2.80	1.107	1.121	1.102	2.929	3.021
15	0.8	60	37.5	4.26	4.97	5.78	1.083	1.086	1.111	4.145	4.109
16	0.6	60	37.5	4.50	5.25	6.12	1.053	1.065	1.079	4.223	4.189
17	1	70	50	0.86	1.01	1.17	1.238	1.219	1.195	2.224	2.362
18	0.6	50	50	0.87	1.01	1.18	1.114	1.125	1.173	2.189	2.301
19	0.8	60	37.5	1.74	2.02	2.35	1.153	1.139	1.131	3.191	3.313

**Table 6:** ANOVA results with authentic replicates only in the central points.

Source	Sum of Squares	df	Mean Square	*F*-Value	*p*-Value
Model	49.89	4	12.47	1150.75	<0.0001
B-Acetonitrile	41.74	1	41.74	3850.76	<0.0001
C-Temperature	2.86	1	2.86	264.07	<0.0001
BC	1.47	1	1.47	135.68	<0.0001
B^2^	3.82	1	3.82	352.50	<0.0001
Residual	0.1517	14	0.0108		
Lack of Fit	0.1438	10	0.0144	7.26	0.0355
Pure Error	0.0079	4	0.0020		
Cor Total	50.04	18			

**Table 7:** Verification run for the chosen working point in the MODR.

Response Variable Name	Experimental Result	Predicted Result	Low 2 Sigma Confidence Limit	High 2 Sigma Confidence Limit
BMC Retention Factor	3.49	3.64	3.38	3.90
DMC Retention Factor	3.97	4.13	3.85	4.40
CUR Retention Factor	4.52	4.67	4.38	4.96
BMC Tailing Factor	1.11	1.09	1.04	1.14
DMC Tailing Factor	1.10	1.10	1.07	1.13
CUR Tailing Factor	1.08	1.09	1.07	1.12
BMC-DMC Resolution	3.15	3.20	3.10	3.30
DMC-CUR Resolution	3.25	3.26	3.17	3.34

## Data Availability

Data are contained within the article and Supplementary Materials.

## Supplementary Materials

The following supporting information can be downloaded at https://www.mdpi.com/article/10.3390/molecules29246057/s1: Figure S1: Diagnostic plots of retention factor DMC from Design Expert software—(a) normality plot; (b) residuals vs. predicted; (c) residuals vs. run. The red line in B and C represents the boundaries of ±3 Studentized residuals, which denotes a 99.7% confidence level. For a 95% confidence level, the points should be within the ±2 Studentized residual limits; Figure S2: (a) Cook’s distance and (b) leverage plots and (c) predicted vs. actual for the retention factor DMC model from Design Expert; Figure S3: Diagnostic plots of the retention factor CUR from Design Expert software—(a) normality plot; (b) residuals vs. predicted; (c) residuals vs. run. The red line in B and C represents the boundaries of ±3 Studentized residuals, which denotes a 99.7% confidence level. For a 95% confidence level, the points should be within the ±2 Studentized residual limits; Figure S4: (a) Cook’s distance and (b) leverage plots and (c) predicted vs. actual for the retention factor CUR model from Design Expert; Figure S5: Diagnostic plots of the tailing BMC from Design Expert software—(a) normality plot; (b) residuals vs. predicted; (c) residuals vs. run. The red line in B and C represents the boundaries of ±3 Studentized residuals, which denotes a 99.7% confidence level. For a 95% confidence level, the points should be within the ±2 Studentized residual limits; Figure S6: (a) Cook’s distance and (b) leverage plots and (c) predicted vs. actual for the tailing BMC model from Design Expert; Figure S7: Diagnostic plots of the tailing DMC from Design Expert software—(a) normality plot; (b) residuals vs. predicted; (c) residuals vs. run. The red line in B and C represents the boundaries of ±3 Studentized residuals, which denotes a 99.7% confidence level. For a 95% confidence level, the points should be within the ±2 Studentized residual limits; Figure S8: (a) Cook’s distance and (b) leverage plots and (c) predicted vs. actual for the tailing DMC model from Design Expert; Figure S9: Diagnostic plots of the tailing CUR from Design Expert software—(a) normality plot; (b) residuals vs. predicted; (c) residuals vs. run. The red line in B and C represents the boundaries of ±3 Studentized residuals, which denotes a 99.7% confidence level. For a 95% confidence level, the points should be within the ±2 Studentized residual limits; Figure S10: (a) Cook’s distance and (b) leverage plots and (c) predicted vs. actual for the tailing CUR model from Design Expert; Figure S11: Diagnostic plots of the resolution (BMC, DMC) from Design Expert software—(a) normality plot; (b) residuals vs. predicted; (c) residuals vs. run. The red line in B and C represents the boundaries of ±3 Studentized residuals, which denotes a 99.7% confidence level. For a 95% confidence level, the points should be within the ±2 Studentized residual limits; Figure S12: (a) Cook’s distance and (b) leverage plots and (c) predicted vs. actual for the resolution (BMC, DMC) model from Design Expert; Figure S13: Diagnostic plots of the resolution (DMC, CUR) from Design Expert software—(a) normality plot; (b) residuals vs. predicted; (c) residuals vs. run. The red line in B and C represents the boundaries of ±3 Studentized residuals, which denotes a 99.7% confidence level. For a 95% confidence level, the points should be within the ±2 Studentized residual limits; Figure S14: (a) Cook’s distance and (b) leverage plots and (c) predicted vs. actual for the resolution (DMC, CUR) model from Design Expert; Figure S15: (a) Counter plot and (b) the 3D mean response surface of the retention factor DMC response; Figure S16: (a) Counter plot and (b) the 3D mean response surface of the retention factor CUR response; Figure S17: (a) Counter plot and (b) the 3D mean response surface of the tailing BMC response; Figure S18: (a) Counter plot and (b) the 3D mean response surface of the tailing DMC response; Figure S19: (a) Counter plot and (b) the 3D mean response surface of the tailing CUR response; Figure S20: (a) Counter plot and (b) the 3D mean response surface of the resolution (BMC, DMC) response; Figure S21: (a) Counter plot and (b) the 3D mean response surface of the resolution (DMC, CUR) response; Table S1. Experimental matrix of the face-centered central composite experimental design, with the responses obtained using a single solution for all runs. Table S2: ANOVA results from Design Expert for the retention factor of DMC, using the data performed with one independent solution the curcuminoids for each run; Table S3: ANOVA results from Design Expert for the retention factor of CUR, using the data performed with one independent solution the curcuminoids for each run; Table S4: ANOVA results from Design Expert for the tailing BMC, using the data performed with one independent solution the curcuminoids for each run; Table S5: ANOVA results from Design Expert for the tailing DMC, using the data performed with one independent solution the curcuminoids for each run; Table S6: ANOVA results from Design Expert for the tailing CUR, using the data performed with one independent solution the curcuminoids for each run; Table S7: ANOVA results from Design Expert for the resolution (BMC, DMC), using the data performed with one independent solution the curcuminoids for each run; Table S8: ANOVA results from Design Expert for the resolution (DMC, CUR), using the data performed with one independent solution the curcuminoids for each run; Table S9: Fit statistics of the models. Table S10: Experimental and predicted results of the verification run from Figure 7 after a prolonged time of column use.

## References

[1] Hibbert D.B. (2012). Experimental Design in Chromatography: A Tutorial Review. J. Chromatogr. B Anal. Technol. Biomed. Life Sci..

[2] Breitkreitz M.C. (2021). Analytical Quality by Design. Braz. J. Anal. Chem..

[3] (2024). General Chapterr <1220> Analytical Procedure Life Cycle.

[4] (2023). ICH Q14 Analytical Procedure Development, International Conference on Harmonization. https://database.ich.org/sites/default/files/ICH_Q14_Guideline_2023_1116_1.pdf.

[5] Peraman R., Bhadraya K., Padmanabha Reddy Y. (2015). Analytical Quality by Design: A Tool for Regulatory Flexibility and Robust Analytics. Int. J. Anal. Chem..

[6] Patel K.G., Patel A.T., Shah P.A., Gandhi T.R. (2017). Multivariate Optimization for Simultaneous Determination of Aspirin and Simvastatin by Reverse Phase Liquid Chromatographic Method Using AQbD Approach. Bull. Fac. Pharm. Cairo Univ..

[7] Breitkreitz M.C., Goicoechea H.C. (2023). Introduction to Quality by Design in Pharmaceutical Manufacturing and Analytical Development.

[8] Rozet E., Lebrun P., Hubert P., Debrus B., Boulanger B. (2013). Design Spaces for Analytical Methods. TrAC-Trends Anal. Chem..

[9] Mahr A.G., Lourenço F.R., Borman P., Weitzel J., Roussel J.-M. (2023). Analytical Target Profile (ATP) and Method Operable Design Region (MODR). Introduction to Quality by Design in Pharmaceutical Manufacturing and Analytical Development.

[10] Vera Candioti L., De Zan M.M., Cámara M.S., Goicoechea H.C. (2014). Experimental Design and Multiple Response Optimization. Using the Desirability Function in Analytical Methods Development. Talanta.

[11] StatEase. https://www.statease.com/docs/v23.0/contents/advanced-topics/propagation-of-error/.

[12] Abreu J.C., Mahr A.G., do Lago C.L. (2021). Stability-Indicating Method Development for Quantification of Bromopride, Its Impurities, and Degradation Products by Ultra-High Performance Liquid Chromatography Applying Analytical Quality by Design Principles. J. Pharm. Biomed. Anal..

[13] Verseput R. (2021). Fusion QbD® Software Implementation of APLM Best Practices for Analytical Method Development, Validation, and Transfer. Optimization in HPLC: Concepts and Strategies.

[14] Deng Q., Liu Y., Liu D., Meng Z., Hao X. (2024). Development of a Design of Experiments (DOE) Assistant Modified QuEChERS Method Coupled with HPLC-MS/MS Simultaneous Determination of Twelve Lipid-Soluble Pesticides and Four Metabolites in Chicken Liver and Pork. J. Food Compos. Anal..

[15] Nompari L., Orlandini S., Pasquini B., Campa C., Rovini M., Del Bubba M., Furlanetto S. (2018). Quality by Design Approach in the Development of an Ultra-High-Performance Liquid Chromatography Method for Bexsero Meningococcal Group B Vaccine. Talanta.

[16] Kurmi M., Jayaraman K., Natarajan S., Kumar G.S., Bhutani H., Bajpai L. (2020). Rapid and Efficient Chiral Method Development for Lamivudine and Tenofovir Disoproxil Fumarate Fixed Dose Combination Using Ultra-High Performance Supercritical Fluid Chromatography: A Design of Experiment Approach. J. Chromatogr. A.

[17] Debrus B., Guillarme D., Rudaz S. (2013). Improved Quality-by-Design Compliant Methodology for Method Development in Reversed-Phase Liquid Chromatography. J. Pharm. Biomed. Anal..

[18] Sen S., Ranjan O.P. (2024). A Quality by Design (QbD) Driven Gradient High Performance Liquid Chromatography Method Development for the Simultaneous Estimation of Dasatinib and Nilotinib in Lipid Nanocarriers. J. Chromatogr. B Anal. Technol. Biomed. Life Sci..

[19] Cernosek T., Jain N., Dalphin M., Behrens S., Wunderli P. (2024). Accelerated Development of a SEC-HPLC Procedure for Purity Analysis of Monoclonal Antibodies Using Design of Experiments. J. Chromatogr. B Anal. Technol. Biomed. Life Sci..

[20] Yabré M., Ferey L., Somé T.I., Sivadier G., Gaudin K. (2020). Development of a Green HPLC Method for the Analysis of Artesunate and Amodiaquine Impurities Using Quality by Design. J. Pharm. Biomed. Anal..

[21] Montgomery D.C., Peck E.A., Vining G.G. (2012). Introduction to Linear Regression Analysis.

[22] Montgomery D.C. (2017). Design and Analysis of Experiments.

[23] Minitab Blog. https://blog.minitab.com/en/adventures-in-statistics-2/multiple-regession-analysis-use-adjusted-r-squared-and-predicted-r-squared-to-include-the-correct-number-of-variables.

[24] Neto B.B., Scarminio I.S., Bruns R.E. (2010). Como Fazer Experimentos.

[25] StatEase. https://www.statease.com/docs/v22.0/contents/analysis/anova-output/.

[26] Fusion QbD Fusion LC Method Development Practical User’s Guide Version 10.1. S-Matrix Corporation 2021. www.smatrix.com.

[27] Myers R.H., Montgomery D.C., Anderson-Cook C.M. (2016). Response Surface Methodology: Process and Product Optimization Using Designed Experiments.

[28] James G., Witten D., Hastie T., Tibshirani R. (2021). An Introduction to Statistical Learning-with Applications in R.

[29] Montgomery D.C., Myers R.H., Carter W.H., Vining G.G. (2005). The Hierarchy Principle in Designed Industrial Experiments. Qual. Reliab. Eng. Int..

[30] Peixoto J.L. (1987). Hierarchical Variable Selection in Polynomial Regression Models. Am. Stat..

[31] Peixoto J.L. (1990). A Property of Well-Formulated Polynomial Regression Models. Am. Stat..

[32] Vining G. (2011). Technical Advice: Residual Plots to Check Assumptions. Qual. Eng..

[33] StatEase. https://www.statease.com/docs/v22.0/contents/analysis/diagnostics/diagnostics-plots/#diagnostics-plots.

[34] StatEase. https://www.statease.com/docs/v22.0/contents/analysis/diagnostics/diagnostics-report/#diagnostics-report.

[35] StatEase. https://www.statease.com/docs/v22.0/screen-tips/analysis-node/diagnostics/predicted-vs-actual/.

[36] StatEase. https://www.statease.com/docs/v22.0/contents/analysis/diagnostics/influence-plots/#influence-plots.

[37] Bouabidi A., Ziemons E., Marini R., Hubert C., Talbi M., Bouklouze A., Bourichi H., El Karbane M., Boulanger B., Hubert P. (2012). Usefulness of Capability Indices in the Framework of Analytical Methods Validation. Anal. Chim. Acta.

[38] StatEase. https://www.statease.com/blog/adding-intervals-optimization-graphs/.

[39] StatEase. https://www.statease.com/docs/latest/contents/analysis/interval-estimate-calculation/#interval-estimate-calculation.

