# Supplementary material for: Important Aspects of the Design of Experiments and Data Treatment in the Analytical Quality by Design Framework for Chromatographic Method Development
Source: Molecules. 2024 Dec 23;29(24):6057. doi: 10.3390/molecules29246057 (PMC11678043; doi:10.3390/molecules29246057)
Supplement: Supplementary file 1 [file molecules-29-06057-s001.zip › molecules-3320720-supplementary.pdf]

Supplementary Material

Table S1. Experimental matrix of the face-centered central composite experimental design with the responses obtained using a single solution for all runs.

Run	Factor			Response							
	A	B	C	k BMC	k DMC	k CUR	Tailing BMC	Tailing DMC	Tailing CUR	Rs (BMC, DMC)	Rs (DMC, CUR)
1	0.6	50	25	6.06	6.78	7.56	1.065	1.070	1.063	3.156	3.181
2	0.8	50	37.5	4.93	5.66	6.49	1.102	1.090	1.074	3.805	3.866
3	0.6	70	50	0.78	0.91	1.07	1.334	1.281	1.239	2.174	2.341
4	1	60	37.5	1.96	2.25	2.58	1.164	1.153	1.127	2.648	2.756
5	0.8	60	37.5	1.97	2.26	2.60	1.169	1.159	1.131	2.829	2.947
6	0.8	60	37.5	1.96	2.26	2.59	1.166	1.157	1.128	2.832	2.947
7	0.8	60	37.5	1.97	2.26	2.59	1.167	1.157	1.13	2.833	2.944
8	0.6	70	25	1.09	1.23	1.39	1.254	1.232	1.198	1.904	2.007
9	1	50	50	4.03	4.74	5.57	1.123	1.093	1.078	4.338	4.435
10	0.8	60	50	1.65	1.93	2.25	1.212	1.179	1.146	3.182	3.340
11	1	50	25	6.02	6.77	7.60	1.050	1.049	1.049	2.694	2.750
12	1	70	25	1.07	1.22	1.38	1.215	1.188	1.156	1.594	1.682
13	0.8	70	37.5	0.91	1.05	1.21	1.265	1.239	1.208	1.946	2.071
14	0.8	60	25	2.35	2.64	2.97	1.150	1.127	1.114	2.356	2.432
15	0.8	60	37.5	1.96	2.26	2.59	1.172	1.158	1.132	2.833	2.944
16	0.6	60	37.5	1.99	2.28	2.61	1.180	1.158	1.131	2.962	3.078
17	1	70	50	0.77	0.91	1.06	1.340	1.289	1.249	1.970	2.115
18	0.6	50	50	4.01	4.72	5.53	1.088	1.055	1.068	4.537	4.611
19	0.8	60	37.5	1.97	2.27	2.60	1.166	1.157	1.129	2.825	2.938

Table S2. ANOVA results from Design Expert for the retention factor of DMC, using the data performed with one independent solution the curcuminoids for each run.

Source	Sum of Squares	df	Mean Square	F-value	p-value	
**Model**	74,07	4	18,52	1242,84	< 0.0001	significant
B-Acetonitrile	63,20	1	63,20	4241,74	< 0.0001	
C-Temperature	2,72	1	2,72	182,88	< 0.0001	
BC	1,51	1	1,51	101,60	< 0.0001	
B^2	6,63	1	6,63	445,16	< 0.0001	
**Residual**	0,2086	14	0,0149			
Lack of Fit	0,1987	10	0,0199	8,01	0,0298	significant
Pure Error	0,0099	4	0,0025			
**Cor Total**	74,28	18				

Table S3. ANOVA results from Design Expert for the retention factor of CUR, using the data performed with one independent solution the curcuminoids for each run.

Source	Sum of Squares	df	Mean Square	F-value	p-value	
**Model**	93,90	4	23,48	1387,12	< 0.0001	significant
B-Acetonitrile	81,23	1	81,23	4799,46	< 0.0001	
C-Temperature	2,65	1	2,65	156,72	< 0.0001	
BC	1,47	1	1,47	86,90	< 0.0001	
B^2	8,55	1	8,55	505,41	< 0.0001	
**Residual**	0,2369	14	0,0169			
Lack of Fit	0,2243	10	0,0224	7,07	0,0372	significant
Pure Error	0,0127	4	0,0032			
**Cor Total**	94,14	18				

Table S4. ANOVA results from Design Expert for the Tailing BMC, using the data performed with one independent solution the curcuminoids for each run.

Source	Sum of Squares	df	Mean Square	F-value	p-value	
**Model**	0,0300	3	0,0100	19,34	< 0.0001	significant
A-Flow rate	0,0044	1	0,0044	8,45	0,0109	
B-Acetonitrile	0,0230	1	0,0230	44,56	< 0.0001	
AB	0,0026	1	0,0026	5,01	0,0407	
**Residual**	0,0078	15	0,0005			
Lack of Fit	0,0070	11	0,0006	3,30	0,1303	not significant
Pure Error	0,0008	4	0,0002			
**Cor Total**	0,0378	18				

Table S5. ANOVA results from Design Expert for the Tailing DMC, using the data performed with one independent solution the curcuminoids for each run.

Source	Sum of Squares	df	Mean Square	F-value	p-value	
**Model**	0,0201	3	0,0067	29,92	< 0.0001	significant
A-Flow rate	0,0036	1	0,0036	15,96	0,0012	
B-Acetonitrile	0,0150	1	0,0150	66,91	< 0.0001	
AB	0,0015	1	0,0015	6,88	0,0192	
**Residual**	0,0034	15	0,0002			
Lack of Fit	0,0030	11	0,0003	3,44	0,1222	not significant
Pure Error	0,0003	4	0,0001			
**Cor Total**	0,0234	18				

Table S6. ANOVA results from Design Expert for the Tailing CUR, using the data performed with one independent solution the curcuminoids for each run.

Source	Sum of Squares	df	Mean Square	F-value	p-value	
**Model**	0,0182	5	0,0036	28,97	< 0.0001	significant
A-Flow rate	0,0016	1	0,0016	12,84	0,0033	
B-Acetonitrile	0,0092	1	0,0092	73,58	< 0.0001	
C-Temperature	0,0011	1	0,0011	9,12	0,0099	
BC	0,0014	1	0,0014	11,39	0,0050	
B^2	0,0048	1	0,0048	37,93	< 0.0001	
**Residual**	0,0016	13	0,0001			
Lack of Fit	0,0012	9	0,0001	1,25	0,4439	not significant
Pure Error	0,0004	4	0,0001			
**Cor Total**	0,0198	18				

Table S7. ANOVA results from Design Expert for the resolution (BMC, DMC), using the data performed with one independent solution the curcuminoids for each run.

Source	Sum of Squares	df	Mean Square	F-value	p-value	
**Model**	8,04	6	1,34	701,42	< 0.0001	significant
A-Flow rate	0,0155	1	0,0155	8,13	0,0146	
B-Acetonitrile	6,22	1	6,22	3255,12	< 0.0001	
C-Temperature	1,39	1	1,39	725,87	< 0.0001	
AC	0,0204	1	0,0204	10,68	0,0067	
BC	0,3612	1	0,3612	189,18	< 0.0001	
C^2	0,0372	1	0,0372	19,51	0,0008	
**Residual**	0,0229	12	0,0019			
Lack of Fit	0,0214	8	0,0027	7,03	0,0385	significant
Pure Error	0,0015	4	0,0004			
**Cor Total**	8,06	18				

Table S8. ANOVA results from Design Expert for the resolution (DMC, CUR), using the data performed with one independent solution the curcuminoids for each run.

Source	Sum of Squares	df	Mean Square	F-value	p-value	
**Model**	7,03	7	1,00	700,91	< 0.0001	significant
A-Flow rate	0,0127	1	0,0127	8,90	0,0125	
B-Acetonitrile	5,28	1	5,28	3689,36	< 0.0001	
C-Temperature	1,35	1	1,35	939,42	< 0.0001	
AC	0,0136	1	0,0136	9,50	0,0104	
BC	0,3003	1	0,3003	209,69	< 0.0001	
B^2	0,0168	1	0,0168	11,76	0,0056	
C^2	0,0132	1	0,0132	9,18	0,0114	
**Residual**	0,0158	11	0,0014			
Lack of Fit	0,0152	7	0,0022	15,09	0,0099	significant

Pure Error	0,0006	4	0,0001
**Cor Total**	7,04	18	

Table S9. Fit statistics of the models.

**Model**	**k DMC**	**k BMC**	**Tailing BMC**	**Tailing DMC**	**Tailing CUR**	**Rs (BMC, DMC)**	**Rs (DMC, CUR)**
R^2	0,9972	0,9975	0,7946	0,8568	0,9176	0,9972	0,9978
Adjusted-R^2	0,9964	0,9968	0,7535	0,8282	0,8860	0,9957	0,9963
Predicted-R^2	0,9933	0,9940	0,6497	0,7615	0,8259	0,9865	0,9863

Table S10. Experimental and predicted results of the verification run from Figure 7 after a prolonged time of column use.

Point	Response Variable Name	Experimental Result	Predicted Result	Low 2 Sigma Confidence Limit	High 2 Sigma Confidence Limit
A	BMC Retention Factor	4.62	4.82	4.56	5.09
	DMC Retention Factor	5.23	5.43	5.16	5.72
	CUR Retention Factor	5.91	6.11	5.82	6.41
	BMC Tailing Factor	1.22	1.08	1.03	1.13
	DMC Tailing Factor	1.21	1.09	1.05	1.13
	CUR Tailing Factor	1.22	1.10	1.07	1.12
	BMC-DMC Resolution	2.61	3.22	3.12	3.32
	DMC-CUR Resolution	2.69	3.25	3.16	3.34
B	BMC Retention Factor	2.86	3.02	2.76	3.29
	DMC Retention Factor	3.23	3.40	3.13	3.68
	CUR Retention Factor	3.66	3.83	3.53	4.12
	BMC Tailing Factor	1.26	1.10	1.05	1.15
	DMC Tailing Factor	1.25	1.11	1.08	1.14
	CUR Tailing Factor	1.23	1.09	1.07	1.12
	BMC-DMC Resolution	2.24	2.87	2.76	2.96
	DMC-CUR Resolution	2.38	2.96	2.87	3.05
T	BMC Retention Factor	3.43	3.64	3.38	3.90
	DMC Retention Factor	3.91	4.13	3.85	4.40
	CUR Retention Factor	4.44	4.67	4.38	4.96
	BMC Tailing Factor	1.26	1.09	1.04	1.14
	DMC Tailing Factor	1.25	1.10	1.07	1.13
	CUR Tailing Factor	1.24	1.09	1.07	1.12

	BMC-DMC Resolution	2.56	3.20	3.10	3.30
	DMC-CUR Resolution	2.68	3.26	3.17	3.34
C	BMC Retention Factor	4.05	4.34	4.08	4.60
	DMC Retention Factor	4.65	4.95	4.67	5.22
	CUR Retention Factor	5.32	5.63	5.34	5.92
	BMC Tailing Factor	1.29	1.07	1.03	1.13
	DMC Tailing Factor	1.30	1.09	1.06	1.13
	CUR Tailing Factor	1.30	1.10	1.07	1.12
	BMC-DMC Resolution	2.94	3.52	3.45	3.65
	DMC-CUR Resolution	2.99	3.56	3.47	4.69
D	BMC Retention Factor	2.60	2.66	2.41	2.92
	DMC Retention Factor	2.97	3.04	2.77	3.31
	CUR Retention Factor	3.40	3.47	3.18	3.76
	BMC Tailing Factor	1.35	1.10	1.05	1.15
	DMC Tailing Factor	1.30	1.11	1.08	1.14
	CUR Tailing Factor	1.26	1.10	1.07	1.12
	BMC-DMC Resolution	2.44	3.13	3.03	3.23
	DMC-CUR Resolution	2.61	3.21	3.12	3.30

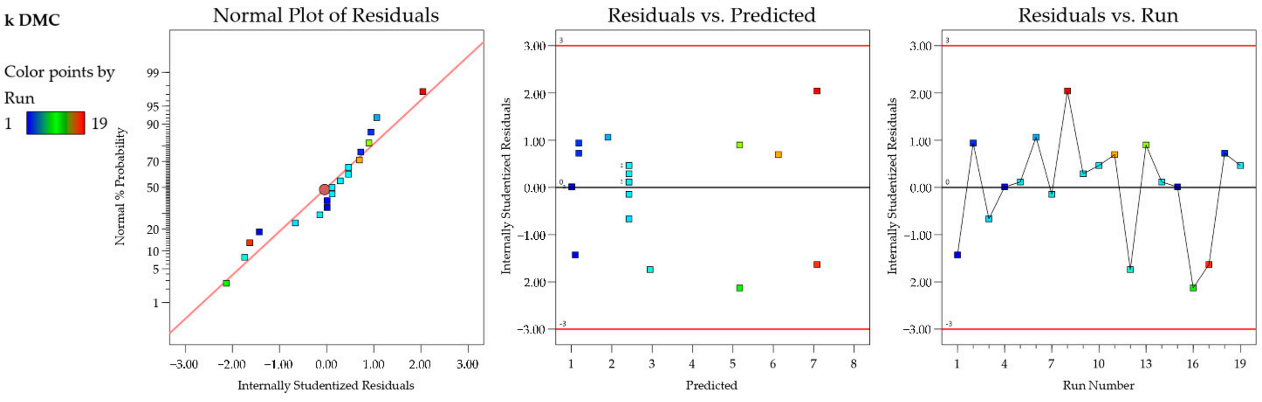

(a) **(b)** **(c)**

Figure S1. Diagnostic plots of the retention factor DMC from Design Expert software – (a) Normality plot; (b) Residuals vs. Predicted; (c) Residuals vs. Run. The red line in B and C represents the boundaries of ± 3 studentized residues which denotes a 99,7% confidence level. For a 95% confidence level, the points should be within the ± 2 studentized residues limits.

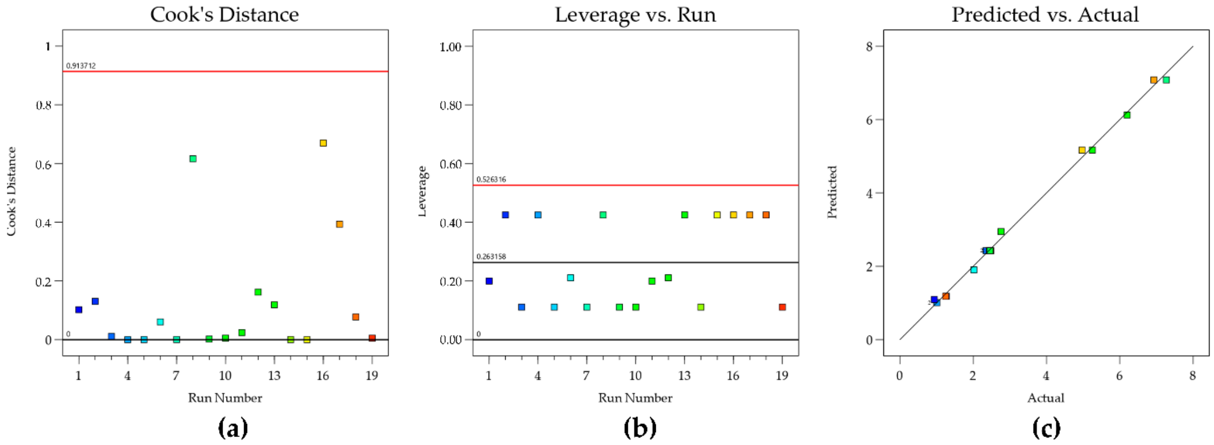

Figure S2. (a) Cook's distance and (b) leverage plots, and (c) predicted vs actual for the retention factor DMC model from Design Expert.

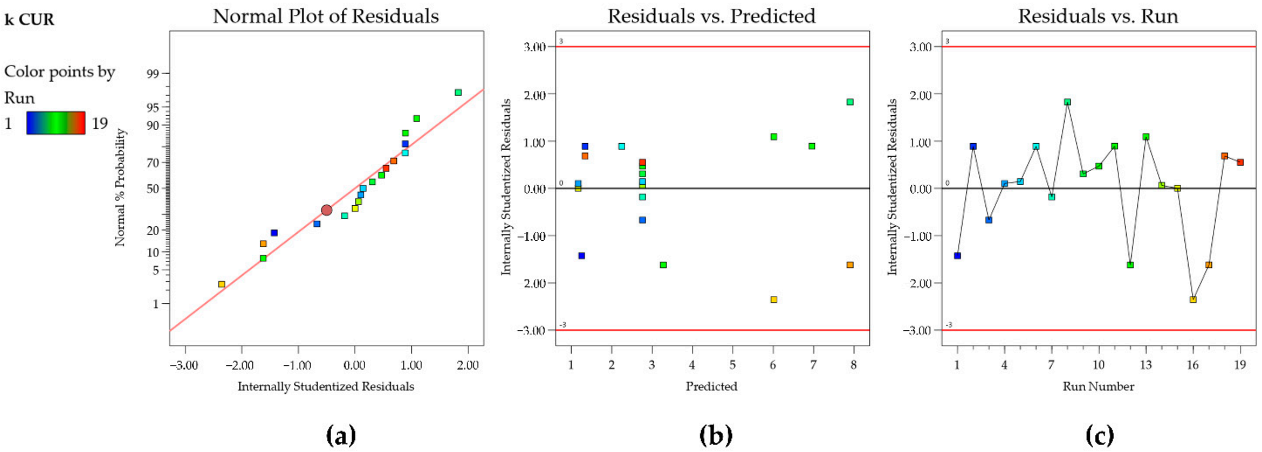

Figure S3. Diagnostic plots of the retention factor CUR from Design Expert software – (a) Normality plot; (b) Residuals vs. Predicted; (c) Residuals vs. Run. The red line in B and C represents the boundaries of ± 3 studentized residues which denotes a 99,7% confidence level. For a 95% confidence level, the points should be within the ± 2 studentized residues limits.

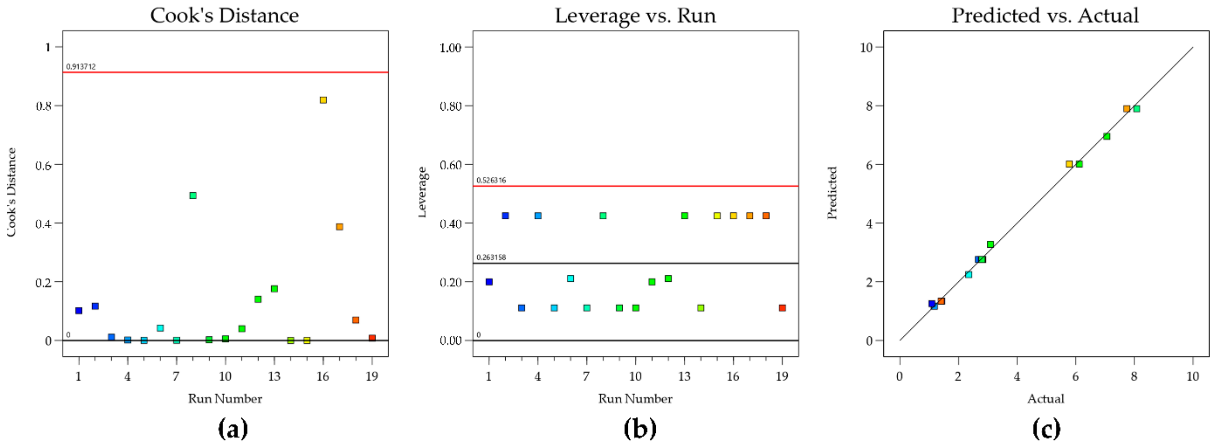

Figure S4. (a) Cook's distance and (b) leverage plots, and (c) predicted vs actual for the retention factor CUR model from Design Expert.

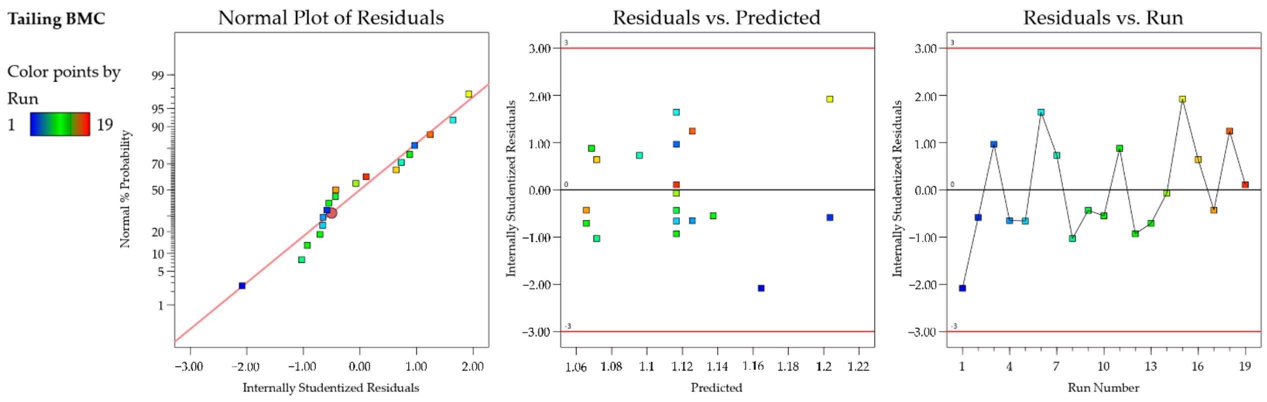

(a) (b) (c)

Figure S5. Diagnostic plots of the tailing BMC from Design Expert software – (a) Normality plot; (b) Residuals vs. Predicted; (c) Residuals vs. Run. The red line in B and C represents the boundaries of ± 3 studentized residues which denotes a 99,7% confidence level. For a 95% confidence level, the points should be within the ± 2 studentized residues limits.

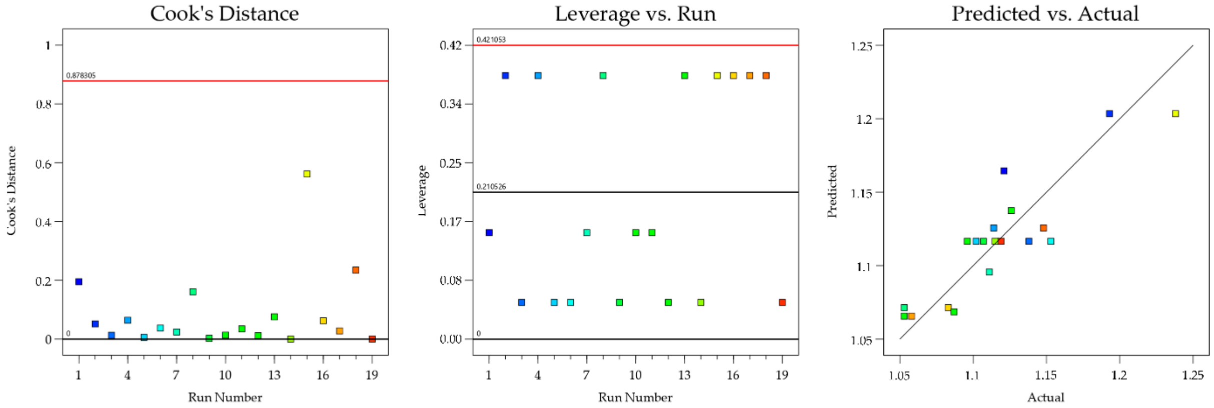

(a) (b) (c)

Figure S6. (a) Cook's distance and (b) leverage plots, and (c) predicted vs actual for the tailing BMC model from Design Expert.

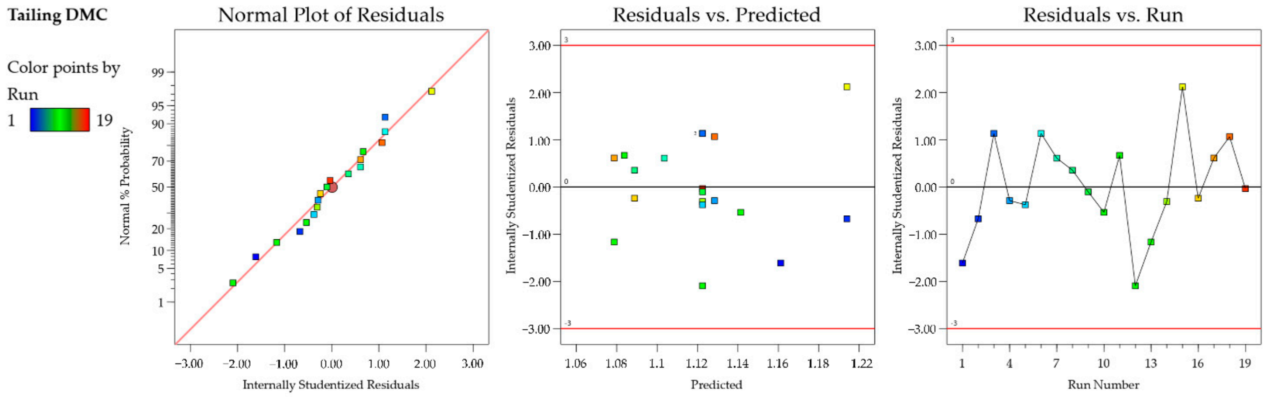

(a) (b) (c)

Figure S7. Diagnostic plots of the tailing DMC from Design Expert software – (a) Normality plot; (b) Residuals vs. Predicted; (c) Residuals vs. Run. The red line in B and C represents the boundaries of ± 3 studentized residues which denotes a 99,7% confidence level. For a 95% confidence level, the points should be within the ± 2 studentized residues limits.

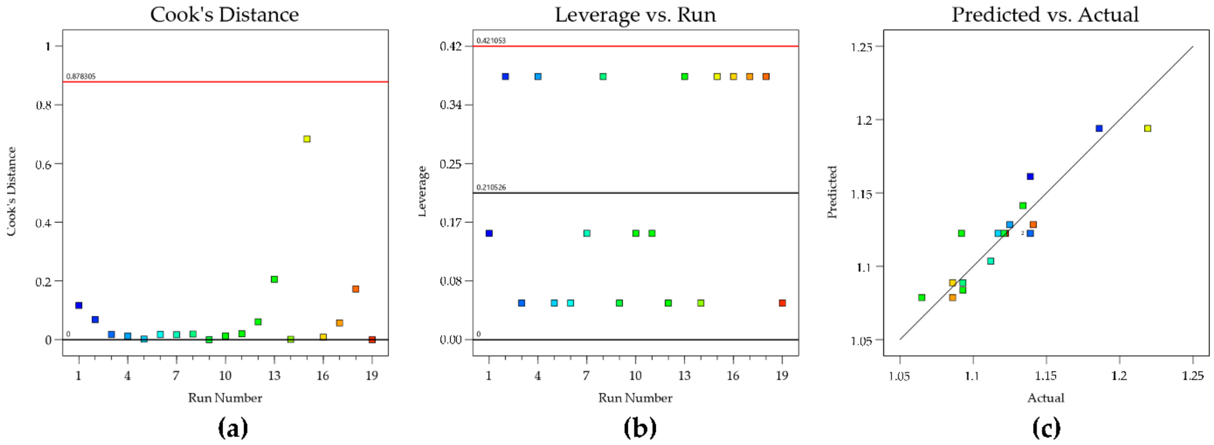

Figure S8. (a) Cook's distance and (b) leverage plots, and (c) predicted vs actual for the tailing DMC model from Design Expert.

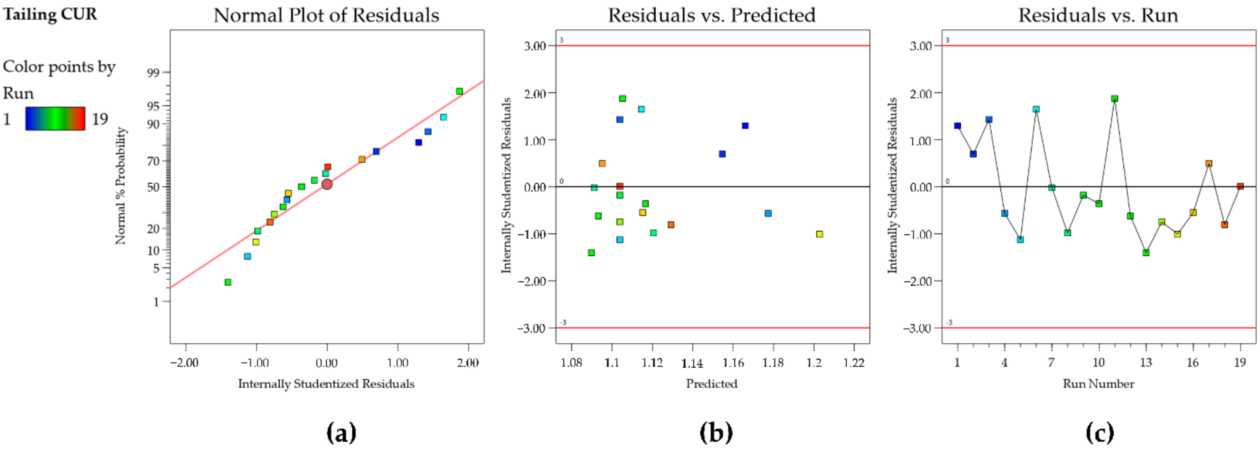

Figure S9. Diagnostic plots of the tailing CUR from Design Expert software – (a) Normality plot; (b) Residuals vs. Predicted; (c) Residuals vs. Run. The red line in B and C represents the boundaries of ± 3 studentized residues which denotes a 99,7% confidence level. For a 95% confidence level, the points should be within the ± 2 studentized residues limits.

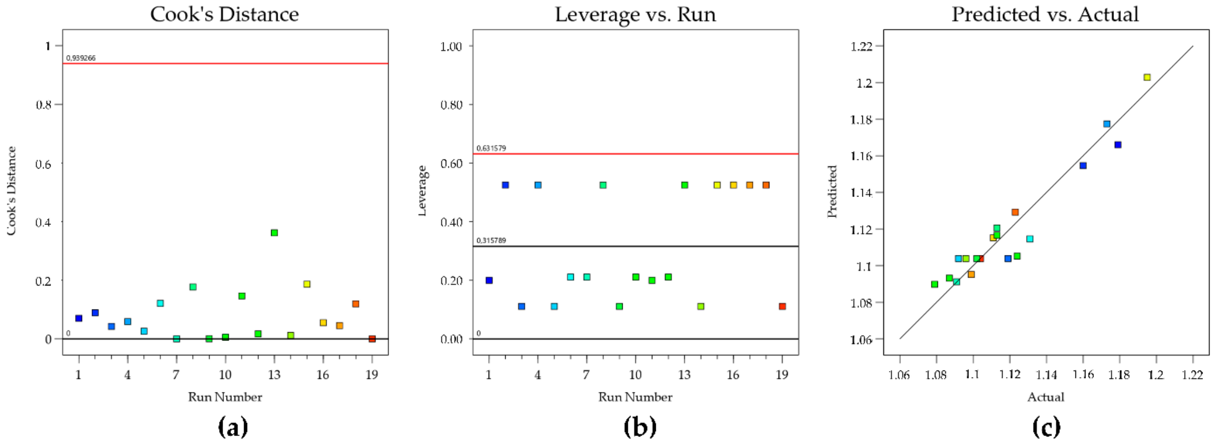

Figure S10. (a) Cook's distance and (b) leverage plots, and (c) predicted vs actual for the tailing CUR model from Design Expert.

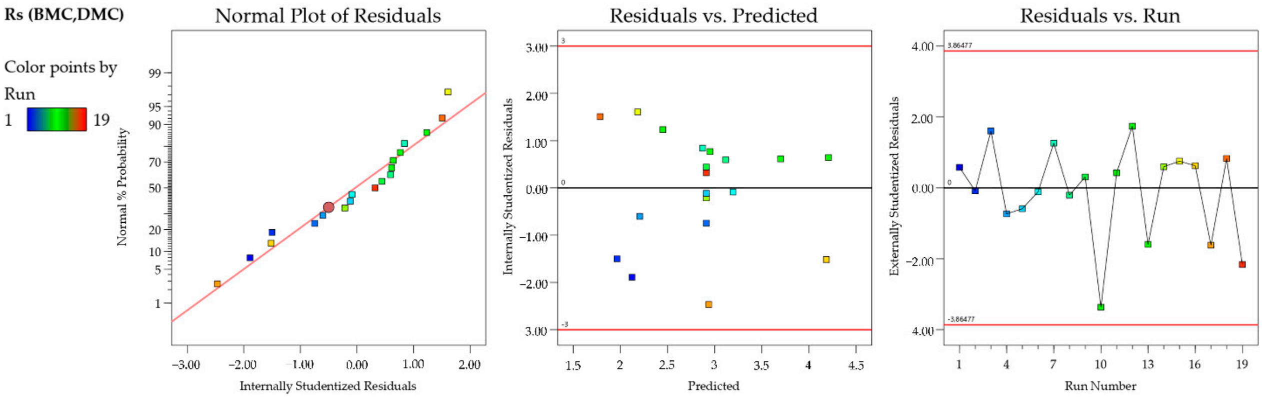

(a) **(b)** **(c)**

Figure S11. Diagnostic plots of the resolution (BMC, DMC) from Design Expert software – (a) Normality plot; (b) Residuals vs. Predicted; (c) Residuals vs. Run. The red line in B and C represents the boundaries of ± 3 studentized residues which denotes a 99,7% confidence level. For a 95% confidence level, the points should be within the ± 2 studentized residues limits.

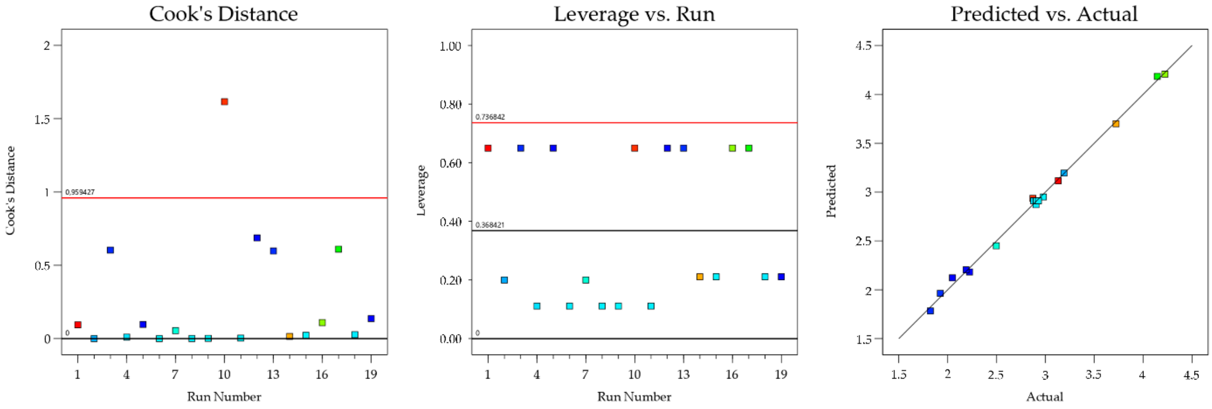

(a) **(b)** **(c)**

Figure S12. (a) Cook's distance and (b) leverage plots, and (c) predicted vs actual for the resolution (BMC, DMC) model from Design Expert.

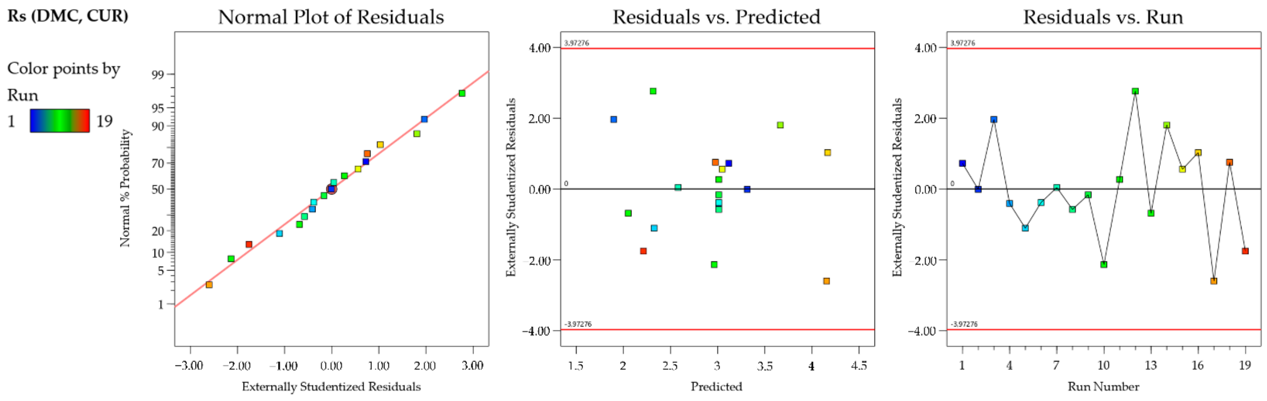

(a) (b) (c)

Figure S13. Diagnostic plots of the resolution (DMC, CUR) from Design Expert software – (a) Normality plot; (b) Residuals vs. Predicted; (c) Residuals vs. Run. The red line in B and C represents the boundaries of ± 3 studentized residues which denotes a 99,7% confidence level. For a 95% confidence level, the points should be within the ± 2 studentized residues limits.

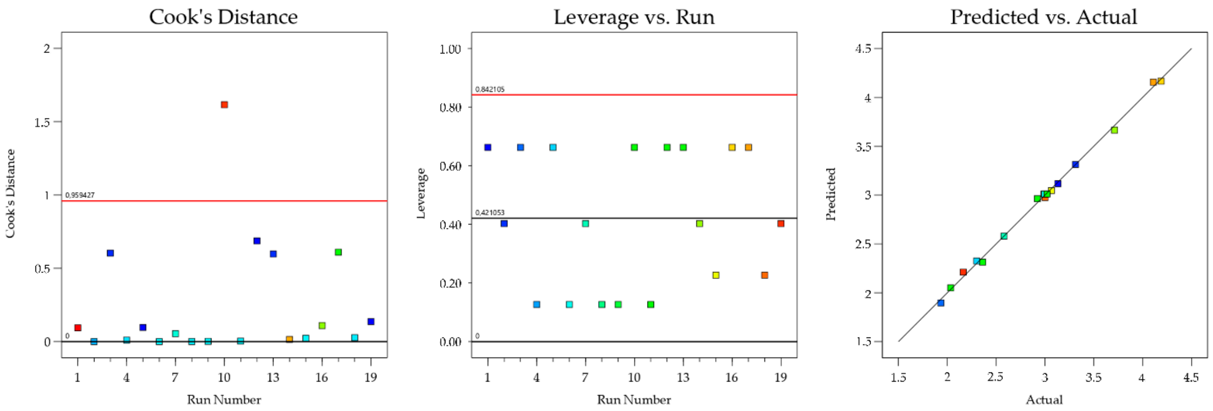

(a) (b) (c)

Figure S14. (a) Cook's distance and (b) leverage plots, and (c) predicted vs actual for the resolution (DMC, CUR) model from Design Expert.

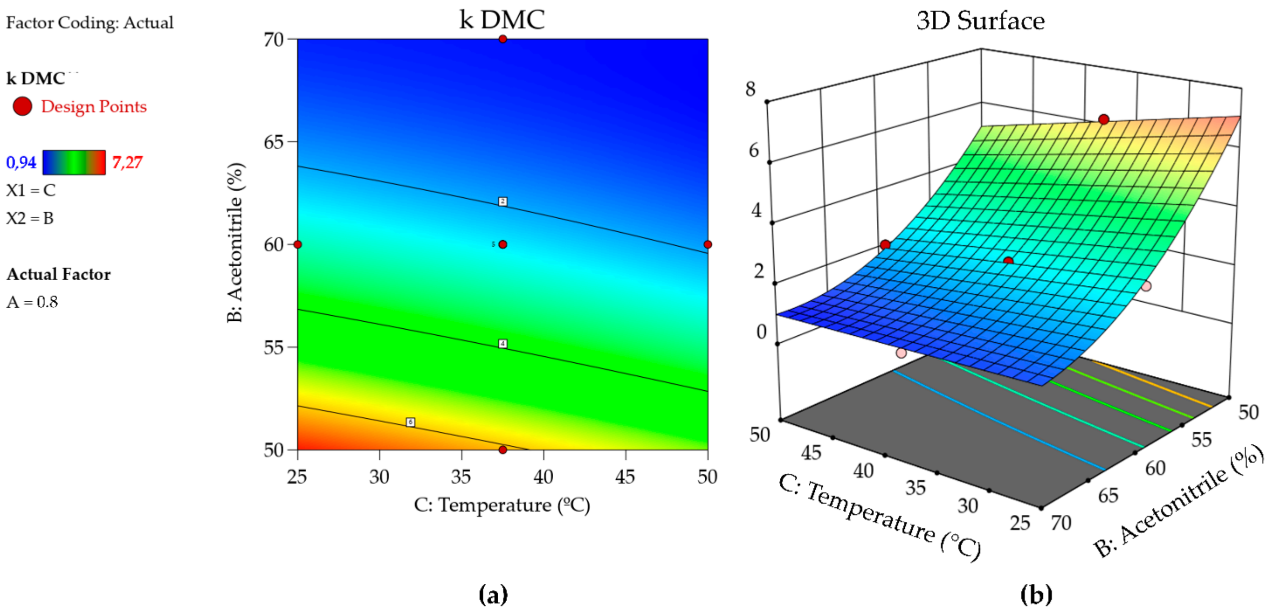

Figure S15. (a) Counter plot and (b) the response surface of the retention factor DMC response.

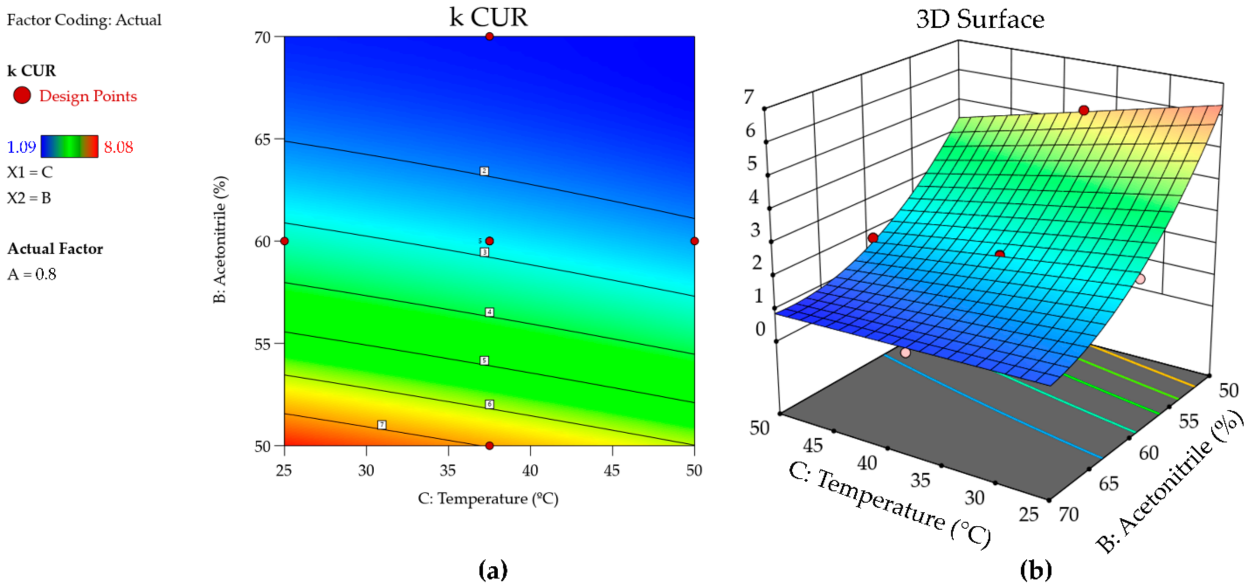

Figure S16. (a) Counter plot and (b) the response surface of the retention factor CUR response.

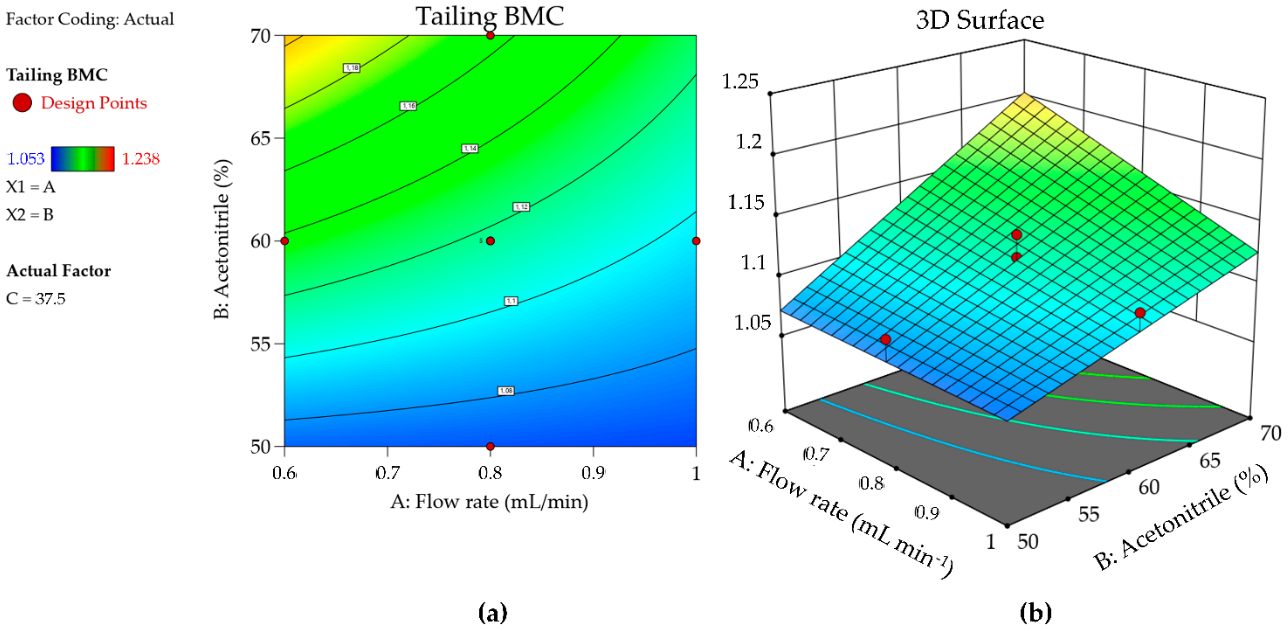

Figure S17. (a) Counter plot and (b) the response surface of the tailing BMC response.

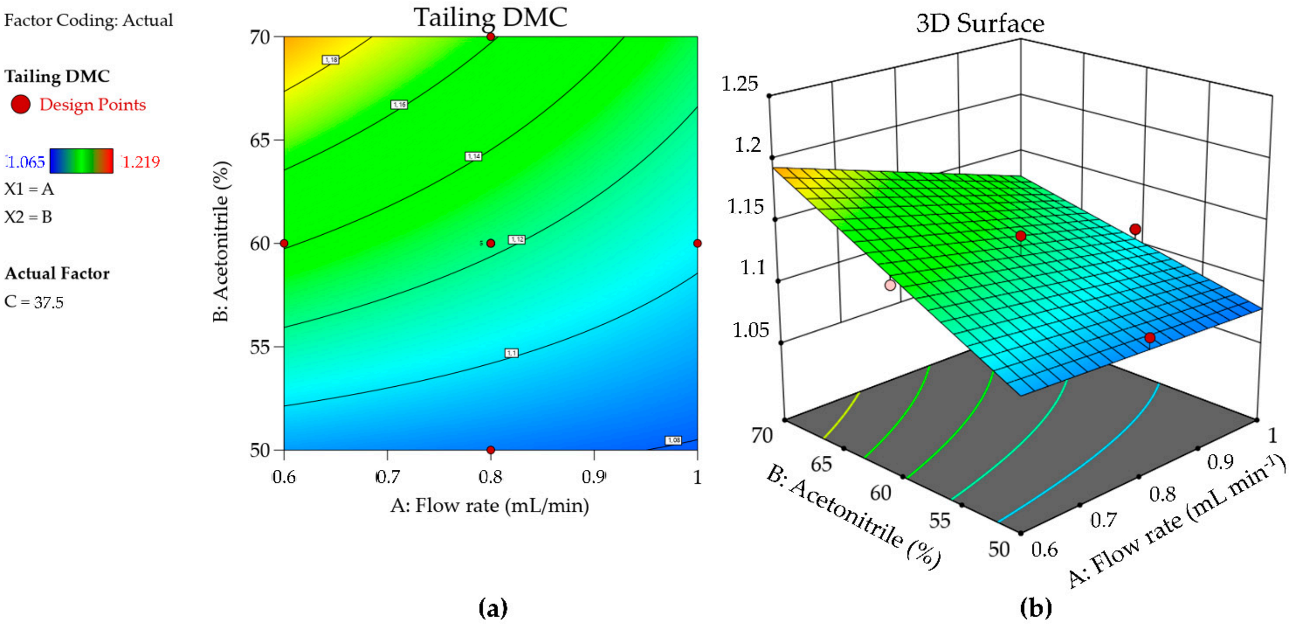

Figure S18. (a) Counter plot and (b) the response surface of the tailing DMC response.

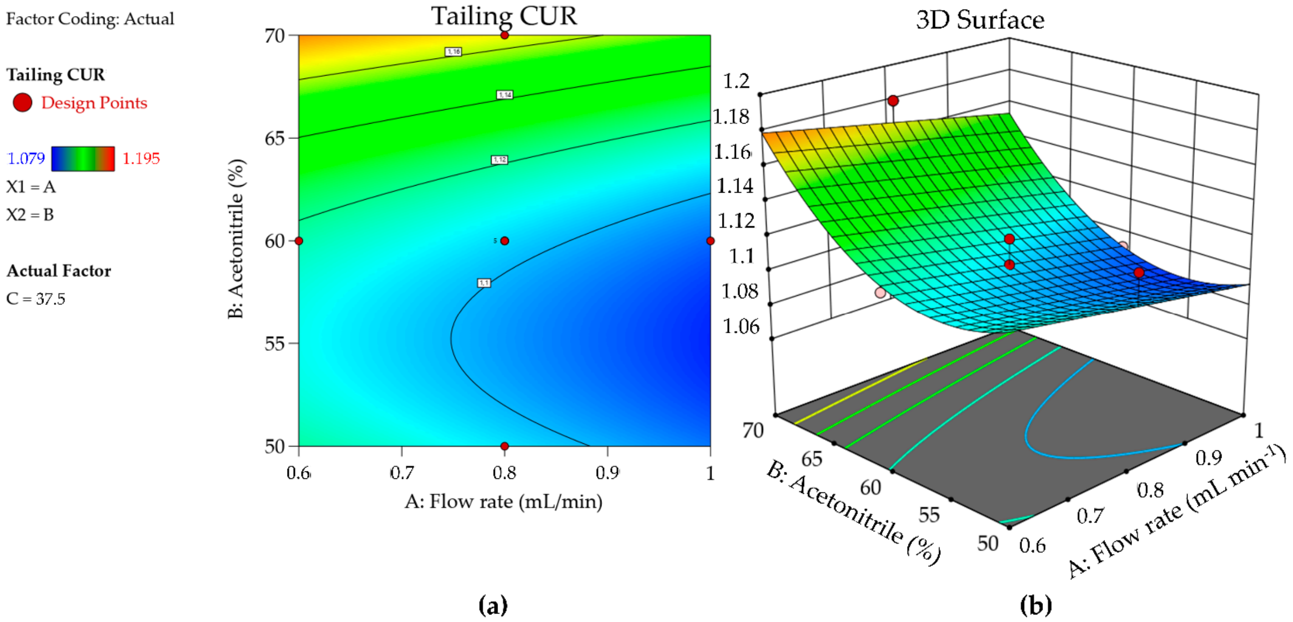

Figure S19. (a) Counter plot and (b) the response surface of the tailing CUR response.

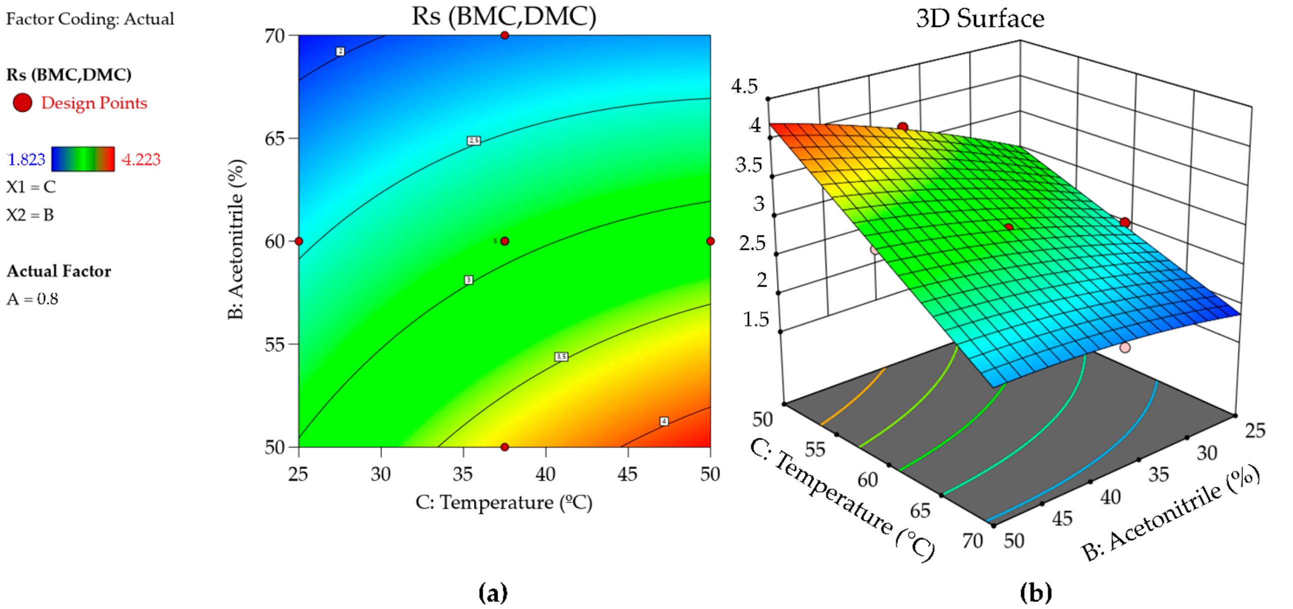

Figure S20. (a) Counter plot and (b) the response surface of the resolution (BMC, DMC) response.

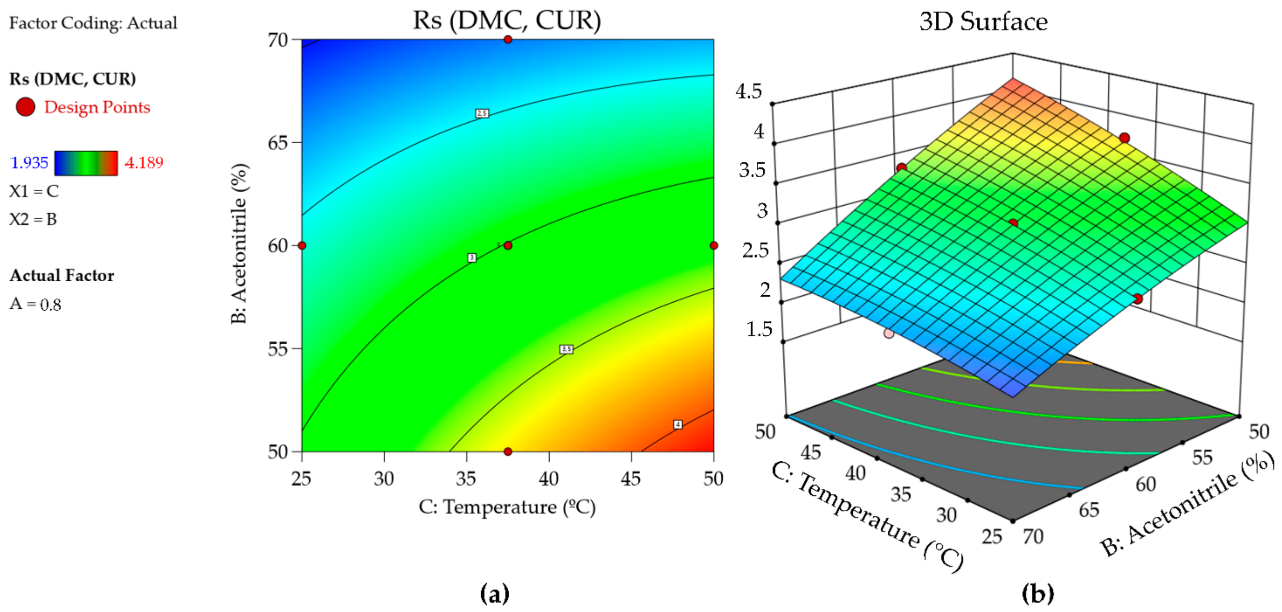

Figure S21. (a) Counter plot and (b) the response surface of the resolution (DMC, CUR) response.
